# Binocular fusion disorders impair basic visual processing

**DOI:** 10.1038/s41598-022-16458-y

**Published:** 2022-07-22

**Authors:** Laura Benhaim-Sitbon, Maria Lev, Uri Polat

**Affiliations:** School of Optometry and Vision Sciences, Faculty of Life Sciences, Bar-Ilan University, Ramat-Gan, Israel

**Keywords:** Neuroscience, Sensory processing

## Abstract

In an era of increasing screen consumption, the requirement for binocular vision is demanding, leading to the emergence of syndromes such as the computer vision syndrome (CVS) or visual discomfort reported by virtual reality (VR) users. Heterophoria (phoria) is a latent eye misalignment (with a prevalence up to 35%) that appears in conditions that disrupt binocular vision and may affect the quality of binocular fusion. Collinear facilitation (CF), the mechanism for grouping contour elements, is a process that reveals lateral interactions by improving the visibility of a target by flankers placed collinearly. An abnormal pattern of CF has been observed in strabismic amblyopia. We hypothesize that phoria may affect CF in the horizontal meridian (HM) due to latent eye misalignment and its impact on binocular fusion. Fully corrected participants (phoria group and controls) completed a standard CF experiment for horizontal and vertical meridians during binocular and monocular viewing. Phoric observers exhibited (1) an asymmetry and an abnormal pattern of CF only for the HM, during both monocular and binocular viewing, (2) poor binocular summation between the monocular inputs, and (3) no binocular advantage of the CF. Phoria affects the CF in a way that is reminiscent of meridional amblyopia without being attributed to abnormal refraction. The abnormal pattern of CF in monocular viewing suggests that phoria could be a binocular developmental disorder that affects monocular spatial interactions. We suggest that the results could contribute to explain the visual discomfort experienced with VR users or symptoms when presenting CVS.

## Introduction

In an era of continuously increasing screen consumption, the requirement for quality binocular vision is quite demanding. Either at work with the use of computers for writing, reading, or virtual meetings or for recreational purposes with smartphones and virtual reality (VR) devices, the average screen time is globally increasing, spiking to more than 13 h a day during lockdowns^1^. This led to the emergence of new syndromes like the computer vision syndrome (CVS) or to visual discomfort and pain reported with the use of head-mounted displays (HMDs) for VR^2^. CVS is defined by the American Optometrist Association as a group of eye-related and vision discomfort conditions appearing with the prolonged use of display devices^3^, which could be explained by heterophoria. The prevalence of CVS ranges from 64 to 75% of computer users^4–6^. Some early studies estimate that 90% of U.S. workers who use computers for more than 3 h per day experience it in some form^7^. It is a growing public health concern because global data show that 60 million people are suffering from CVS and one million new cases occur each year^4^. This contributes significantly to reducing the quality of life and efficiency at the workplace^4,7,8^. As for the VR market, it is expected to reach 18.9 billion US dollars in entertainment by 2025, with the gaming market accounting for one-third of all VR content^9^. Visually induced motion sickness (VIMS) is often encountered due to the use of HMDs and can be due to multiple sensory input integration problems but also to the imbalance of the visual system consecutive to the presence of heterophoria, binocular fusion, or accommodative disorders^2^. Thus, it remains an obstacle to the widespread adoption and commercial development of technologies associated with VR-based HMDs^2^.

Heterophoria is a latent eye misalignment appearing when normal fusion mechanisms are disrupted^10^ (not to be confused with strabismus, i.e., manifest deviation of one or both eyes). Because of the dissociation of the two eyes, the term “dissociated” phoria can also be used. Heterophoria can be horizontal (deviation of the eyes inwards or outwards), vertical (deviation of the eyes upwards or downwards), or cyclorotary (the tendency of the eyes to rotate around their sagittal axis, clockwise or counterclockwise). Heterophoria is a widely used term by vision specialists because its measurement is part of standard binocular vision assessments^11,12^. The overall prevalence of heterophoria in adult populations varies from 12.9%^13^ in young student populations to 35.6% in elderly populations^14^ (up to 38% in old rural populations^15^), (for more details, see the Supplementary Material). Note that heterophoria is not a primary physical property independent of the method of examination (as opposed to, e.g., myopia or manifest strabismus), rather, the angle of phoria is a reaction to an artificial interference with binocular vision.

Binocular vision disorders can result in symptoms that can affect daily visual activities^16^. For example, in reading, efficient binocular vision processes enable a single percept of the text; it has a certain advantage in terms of fixation time and reading speed. This binocular advantage has been shown to be affected by heterophoria; it even appeared that the higher the heterophoria was, the greater was the disadvantage during binocular reading^17^. Untreated heterophoria can be associated with visually induced motion sickness^18^, dyslexia^19^, or postural deficiency syndrome^20^ including chronic back pain. Vertical heterophoria could also be acquired from traumatic brain injury (TBI); its treatment relieves common symptoms associated with TBI^21^. Thus, abnormal values of heterophoria may lead to visual fatigue, migraine^22^, or double vision^11^ and can be involved in CVS^5–7,23,24^. In psychophysics, horizontal phoria was found to disrupt apparent motion perception^25^. Data in our laboratory, taken from high heterophoria subjects, pointed to an atypical behavior, a different pattern of results, and a greater fatigue in the psychophysics experiments on contextual modulation in experiments such as lateral masking and crowding. This led us to hypothesize that heterophoria may affect contextual modulation, especially in the horizontal meridian (HM) due to the latent misalignment of the eyes and its impact on binocular fusion.

Contextual modulation refers to changes in the appearance of target patterns when presented within a surrounding pattern. Various psychophysical phenomena such as perceptual pop-out^26^, contour integration^27–30^, surface perception^31,32^, or figure-ground segregation^33,34^ can account for contextual modulation in the primary visual cortex (V1).

Visual masking is one well-known type of contextual modulation. Here, we will focus on lateral masking, one type of visual masking. Usually, when the target stimulus is presented alone, it is easy for the observer to detect it, but when the target is presented with a mask within a small spatiotemporal window, it can be very difficult. Lateral visual masking experiments reveal lateral interactions in V1. Lateral interaction is the capacity of an excited neuron to either stimulate (facilitate) or inhibit (suppress) the activity of its neighboring neurons. Based on physiological observations, lateral interactions result from a network of long-range connections that exist between similar orientation columns in the visual cortex^35–39^. The lateral masking effect and therefore, lateral interactions are measured as either a decrease (facilitation) or increase (suppression) of low-contrast Gabor patch detection thresholds when flanked by collinearly oriented high-contrast Gabor patches^40,41^.

Facilitation of contrast detection is found when a low-contrast target is presented simultaneously with the maskers^40–48^ or with a delay^49,50^, and it occurs preferentially in collinear configurations^51,52^. A normal pattern of lateral interactions corresponds to a maximal collinear facilitation of target detection for target-flanker separations of 3 wavelengths (λ), a decreased facilitation for longer target-flanker separations, and a suppression for shorter separations^40^. It has been suggested that the size of the receptive fields in V1 is about 2 to 3λ^40,48,49,53,54^.

Collinear facilitation in lateral masking is a mechanism that increases the sharpness and the contrast of the visual response by presenting properties similar to some Gestalt principles of proximity, similarity, good continuation, and closure^27,55,56^. The response to a local element in a contour is modulated by lateral, suppressive, or facilitative inputs, similar to the mechanisms found in collinear facilitation^57^. This suggests that collinear facilitation is the process responsible for grouping^55,58^ and for contour integration^27,59,60^ of elements. Both mechanisms are involved in object recognition in visual perception, although some studies suggest that the collinear facilitation and contour integration mechanisms do not share the same sites^29^.

Lateral masking studies have been investigated mostly under binocular viewing. Visual functions such as visual acuity or contrast sensitivity are known to be enhanced for binocular over monocular viewing: this phenomenon is called binocular summation^61^. Binocular performance (e.g., luminance, visual acuity, and contrast threshold detection) is 40% better than monocular performance^61–68^. Binocular summation is greatest at low contrasts for short presentation times; however, it is reduced with increasing contrast (above 15%) and presentation times^69^. Thus, the interocular suppression is at its lowest for low contrast and short presentation times and increases with contrast^70^. Nevertheless, for the orientation discrimination task, the interocular suppression decreases as the contrast increases ^71^.

Several theories of binocular summation have been elaborated in the past couple of decades^72–74^. According to the gain-control theory^75^, each eye exerts gain control on the other eye in proportion to the strength of its own input^75^. More recently, studies showed conditions in which binocular summation was impaired or abolished^76,77^. Hence, in infantile nystagmus, binocular summation was reduced and could be explained by impaired neural mechanisms of binocular combination (an unequal contribution of the two eyes to binocular summation)^78^. Under crowding conditions, binocular summation was found to be diminished but was restored with tagging (when the target and flankers are ‘‘ungrouped” from each other by making them dissimilar in a fundamental property such as color, polarity, or depth so that the target ‘‘pops-out”)^79^. In the context of suppression by nearby collinear flanking contours, detection of the target is more suppressed under binocular than monocular viewing, thus eliminating the binocular advantage, both at the threshold and suprathreshold levels^77^. However, it has been demonstrated that summation occurs at suprathreshold levels when the effects of suppression are controlled by using a binocular pedestal for both monocular and binocular increments^74^. Nevertheless, a binocular advantage is preserved with more distant flankers up to about four times^77^. No binocular summation of collinear facilitation at a target-to-flanker distance of 3λ was recently found in both controls and subjects that presented oblique astigmatism^76^.

Previous studies of collinear facilitation in neurotypical subjects did not emphasize the differences between monocular and binocular interactions and information regarding the differences between vertical and horizontal meridians (VM and HM, respectively) is lacking^40,56^. However, it was shown that lateral interactions in cases of abnormal binocular experience during the developmental period, for instance in amblyopia, deviate from the normal pattern. Amblyopia is a pathology of binocular vision: it is a unilateral or bilateral decrease in certain visual capabilities, most likely related to the absence of a correlated binocular visual experience during an individual’s critical period of up to his first six to eight years^10,80,81^ with a prevalence of 2–4% in children^82,83^ (for a recent review on the classification, see^84^). An abnormal pattern of lateral interaction was observed in strabismic amblyopia with a suppression at 3λ and in anisometropic amblyopia with a reduced facilitation at 3λ^85^.

Many research studies have been conducted on contextual modulation and heterophoria separately^86^; nevertheless, to date, a study investigating how heterophoria can affect monocular and binocular lateral interactions across the main horizontal and vertical meridians has not yet been published. We hypothesize that heterophoria may affect contextual modulation, especially in the horizontal meridian (HM) due to the latent misalignment of the eyes and its impact on binocular fusion. This research aims at demonstrating how high heterophoria affects collinear facilitation/lateral interactions at a different viewing distance, symmetry (horizontal versus vertical) of lateral interaction, and monocular versus binocular lateral interactions.

We found an abnormal pattern of lateral interactions only in the horizontal meridian (HM) for subjects presenting high horizontal heterophoria at all distances under both binocular and monocular viewing. For the vertical meridian, however, the spatial interactions matched the normal findings in the literature in both groups. We found a strong correlation between the suppression at 3λ and the amount of phoria in absolute values (both are directions of misalignment). In general, we did not find any binocular summation of the lateral interaction for both controls and heterophoric groups; however, we observed greater suppression in binocular viewing for small target-to-flanker separations. Monitoring the eye movement did not reveal any manifest misalignment or statistical difference of eye fixation appearing during the lateral masking (LM) experiment between the two groups. This atypical pattern of lateral interaction, found only in the horizontal meridian (HM) for heterophoria, is reminiscent of meridional amblyopia without being attributed to abnormal refraction of the subjects.

## Methodology

### Participants

Twenty-six participants in total (high heterophoria and controls) were enrolled in the study after they signed a consent form approved by the Internal Review Board (IRB) of Bar-Ilan University. All experimental protocols were performed following the guidelines provided by the committee approving the experiments. All subjects signed a consent form and received financial compensation for their participation. Subjects were recruited using electronic advertisements and direct recruitment. All participants underwent a complete optometric examination by a qualified optometrist within the last year. Subjects had normal or corrected to normal vision. Each subject was included after a comprehensive orthoptic examination by a certified orthoptist including both sensory and motor examinations described herein. Subjects did not show any clinical signs of accommodative disorders, amblyopia, stereopsis disorders, strabismic problems, or ocular disease (exclusion criteria for both groups). Inclusion criteria for the high heterophoric group were age between 18 and 35 years old, horizontal phoria equal or superior to 6Δ (prism diopters), and/or vertical phoria equal or superior to 2Δ at least at one of the experiment’s viewing distances with no decompensation to intermittent strabismus (asymptomatic heterophoria), normal or corrected-to-normal visual acuity (both monocular and binocular), normal stereoscopic vision (40″) and fusion at all distances. Inclusion criteria for the control group were age between 18 and 35 years old, horizontal phoria strictly inferior to 6Δ, and vertical phoria strictly inferior to 2Δ for all the viewing distances, normal or corrected-to-normal visual acuity (both monocular and binocular), normal stereoscopic vision (40″), and fusion at all distances (see Supplementary Material Table 6).

### Procedures of the orthoptic examinations

The sensory assessment included examination of static visual acuity, the dominant eye test, stereoscopic vision, and fusion. Both monocular and binocular static visual acuity was tested by the ETDRS chart at 4 m and 40 cm. The eye dominance was assessed via the hole-in-a-card method^87^, or through a hole formed by the subjects’ hands. The Titmus® Stereotest was used to determine the stereoscopic vision^88^. The presence of fusion at 4 m, 1 m, and 40 cm was confirmed by the Bagolini striated glasses test^89^. The amplitudes of fusion (for both convergence and divergence) were measured by the step vergence testing method^11,90,91^ using a prism bar. Motor evaluation contained an examination of the motility, conjugate eye movements with their amplitude, and eye misalignment (heterophoria or strabismus). The motility was studied in eight cardinal positions of gaze. Finally, eye misalignment, which is the center of the topic of this research, was measured by two different methods: the Maddox rod test (MRT) and the alternating cover test (CT). For detailed procedures, see Supplementary Material -Measurement of heterophoria—procedures.

### Apparatus

The stimuli were presented using a PC controlled by a NVDIA GTX 710 video card and a BENQ XL 2411 color monitor using in-house-developed software for psychophysical and eye-tracking experiments (PSY) developed by Y.S. Bonneh on a Windows PC^92^. The screen resolution was 1920 × 1080 pixels, and gamma correction was applied.

For the eye-tracker recordings, stimuli were displayed at a distance of one meter on a 24-inch LCD monitor (Eizo Foris fg2421) running at 1920 × 1080 screen resolution and at a 100 Hz refresh rate. Eye movements were recorded using the eye-tracking system (EyeLink 1000 infrared system, SR Research, Ontario, Canada), with a sampling rate of 500 Hz, and placed at a distance of 55-60 cm from the eyes. We used a 35 mm lens, a head, and a chin rest. Under these conditions, the EyeLink system is known to have a spatial resolution of 0.01° and an average accuracy of 0.25° to 0.5°. All recordings and analyses were done binocularly. A standard nine-point calibration was used before each session. All experiments were run in dim light.

### Stimuli

Stimuli were localized gray-level gratings (Gabor patches) with a spatial frequency of 4 cycles per degree (cpd) with equal wavelength (λ = 0.433°) and standard deviation (STD, σ), allowing a minimum of 2 cycles in the Gabor patches. The Gabor patches were modulated from a background luminance of 40 cd/m^2^. The effective size of the monitor screen was 52 by 30 cm.

### Procedure

Experiments were run during either binocular viewing (the two eyes seeing simultaneously) or monocular viewing (one eye was occluded by a transparent diffuser). For a summary of the experimental design, see Supplementary Material Table 7.

#### Experiment 1: Contrast detection for single-target and lateral masking in binocular viewing

This experiment was carried out in two phases (see Supplementary Material Table 7). In the first one (referred to as the Pilot study in Supplementary Material), subjects sat at 1 m and 40 cm from the monitor and were tested at three target-to-flanker distances. After collecting the results of 6 subjects for the heterophoria group and 6 subjects for the control group, we decided to add one viewing distance (60 cm) because this is usually the viewing distance when working on computers and it will more likely reflect the context in which people can suffer from CVS. We also decided to add one more target-to-flanker distance (2λ) to better define the limit of collinear facilitation (referred to as experiment 1). For both the Pilot study and experiment 1, up to eight meetings were needed to collect all the data. Meetings took 1–2 h to run a total of 32 blocks for the Pilot study and 60 blocks for experiment 1, depending on the attention capacity and the fatigability of the subject.

A two-alternative temporal forced-choice paradigm and a 3:1 staircase procedure (converging to 79% correct response) were used to measure the target contrast detection threshold^93^. When ready, subjects activated a trial sequence by pressing the middle button of the mouse, after fixating on a small circle in the center of the screen. Subjects were instructed to maintain their fixation in the center of the monitor and to avoid eye movements during the trials. Each trial sequence consisted of a no-stimulus interval (300 ms with a temporal jitter of 500 ms (0–500 added to 300 with equal distribution), a stimulus presentation (80 ms), another no-stimulus interval (700 + 500 ms), and a second stimulus presentation (80 ms). Only one of the two stimulus presentations contained the target (but both contained the mask in the second condition) and were randomly ordered. The stimulus intervals were denoted by four peripheral high contrast crosses. Subjects had to determine which of the two presentations contained the target by pressing a mouse button (left for the first one and right for the second one). Auditory feedback was given for incorrect responses. Throughout the entire experiment, we used a peripheral lock to limit phoria decompensation during the absence of stimuli (and/or interstimulus interval). The peripheral lock consisted of a square sustaining 15.4° visual degrees horizontally and vertically from the center. Four peripheral crosses denoted the interval (first or second) of the Gabor patch presentation. The peripheral lock, Gabor patch frequencies, and cross sizes were modified accordingly to the viewing distance so that experiments at 1 m and 40 cm were identical; only the viewing distance and the phoria associated with this distance were the parameters that changed.

**Figure 1:**
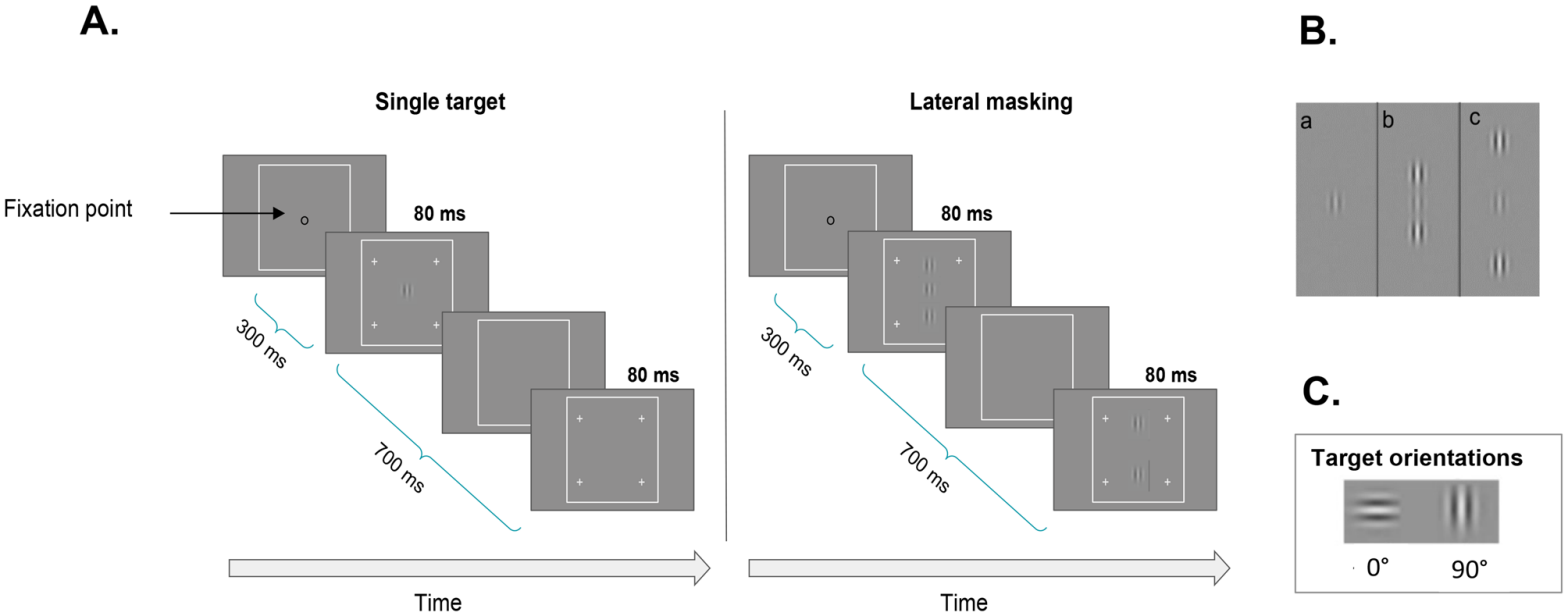
(**A**). Lateral masking paradigm. Participants must decide in which interval (the first or second) the central Gabor target has been presented. The contrast threshold of the target is measured using an adaptive method to reach the 79% correct threshold. (**B)**. Examples of some target-to-flanker distances. (a) Single target, (b) at 3λ, (c) at 6λ. The contrast of the target was enhanced for demonstration purposes. (**C**). Illustrations of the single-target orientations.

We used a standard contrast detection task under lateral masking conditions. The stimuli were presented for 80 ms following two global orientations (0 and 90 degrees, see Fig. 1C). For each orientation, two conditions were tested (Fig. 1A): a single target (T) contrast detection threshold and a contrast detection threshold of T in the presence of two collinear flankers of a contrast of 40% (lateral masking, LM). For experiment 1 (see Supplementary Material Table 7), T and the flankers were separated by 2, 3, 4, and 6λ (units of target wavelength). Thus, for each orientation, the experiments were composed of 5–6 blocks (single target, and LM by 2λ, 3λ, 4λ, and 6λ). Each block consisted of about 65 trials, across which the signal amplitude and the distance between the Gabor signals were kept constant. Screen luminance remained the same during the stimulus and the no-stimulus intervals. Each condition for each orientation was tested at two viewing distances from the monitor: 1 m and 40 cm (the amount of heterophoria can be different if it is at or near the distance viewing). Each experiment was repeated twice so that each subject participated in a total of 4 experiments of lateral masking for each distance.

#### Experiment 2: Contrast detection for single-target and lateral masking in monocular viewing

The experiment took place at 1-m distance from the subjects and only in monocular viewing (both dominant and non-dominant eyes were tested separately). The non-tested eye was occluded by a translucent (frosted) diffuser to avoid binocular rivalry as much as possible. Every other parameter remained the same as in Experiment 1.

#### Experiment 3: Contrast detection for single-target and lateral masking with eye tracker monitoring

We decided to conduct a control experiment to monitor fixation during the LM experiment in binocular viewing. The aim was to verify whether the effect obtained in the heterophoric group was due to a misalignment of the eyes that became manifest (i.e., if an eye turned inwards or outwards during the experiment). The experiment was conducted at a 1-m distance with the same parameters described for Experiment 1. It tested only single-target detection and LM with a target-to-flanker separation of 3λ, where the effect obtained in the LM experiment was more pronounced. The experiment was repeated three times.

### Data and statistical analysis

Repetitions for each presentation and distance condition were averaged for each patient.

Two-way, three-way, and four-way mixed ANOVA were performed to test the effect of 2, 3, or 4 nominal variables (such as group, method, viewing distance, orientation, and separation) on continuous outcomes (threshold elevation or the angle of deviation). Specifically, linear mixed effect models were performed, and the ANOVA was completed on the resulting models. All nominal variables were defined as fixed effects, and subject ID was defined as a random effect. All interactions were included in the initial models. In cases of non-significant interactions, models were refitted without these interactions. Post-hoc analysis was performed as pairwise comparisons defined by linear contrasts, and FDR correction was applied to control for multiple testing. When the interactions were removed, post-hoc analysis was performed by averaging over the non-interacting factors. The normality of the residual homogeneity of variance assumptions was checked graphically.

Pearson’s correlation tests were conducted between threshold elevation and the angle of deviation for different viewing distances. All data points were confirmed for not being outliers.

We used in-house software (PSY, Y.S. Bonneh) to analyze the fixation of each eye during Experiment 3.

## Results

### Experiment 1: Lateral masking in binocular viewing

Since heterophoria is a binocular fusion disorder that may cause discomfort at several working distances, we decided to test the binocular viewing of each subject for lateral masking at 40 cm (which is usually the reading distance), 60 cm (which is usually the computer-working distance to reflect the context where people can suffer from CVS), and at one meter. After the Pilot study (see the Supplementary Material for detailed results), we also decided to add one more target-to-flanker distance (2λ) in order to better define the limit of collinear facilitation.

#### Participants

A total of 16 subjects (8 in the heterophoric group and 8 in the control group) participated in this experiment. Each subject in both groups presented clinically normal stereoscopic vision (40 arcsec). The average age in the control group was 27.6 ± 4.6 years old (mean ± STD), and in the heterophoric group it was 29.5 ± 5.5 years old (mean ± STD).

Among the 8 subjects enrolled in the heterophoric group, two presented an esophoria and six presented an exophoria.

A three-way ANOVA test was performed to evaluate the effect of group, distance, and methods on the heterophoria measurement. Group (F (1,13) = 21.5355, *p* = 0.0005) and distance (F (2,65) = 30.1755, *p* < 0.0005) had a constant effect on the measurement obtained. As for the Pilot study, we did not observe any effect of the methods when measuring heterophoria. The angle of deviation decreased with the distance in both groups. A post-hoc analysis revealed that the angle of deviation of the heterophoric group obtained by both methods was larger than that of the control group at each distance. For more detailed statistical information about the heterophoria measurement of all the enrolled subjects, see Supplementary Material Table 4.

#### Contrast detection threshold for single targets

We measured the contrast detection thresholds for single targets at 40 cm, 60 cm, and 1-m viewing distance for the two orientations (meridians). Contrast detection thresholds for single targets were not statistically different between the two groups for each distance. The values of the contrast detection thresholds obtained are presented in Supplementary Material Table 5. We performed a three-way ANOVA to determine the effect of orientation, viewing distance, and the group on the contrast detection threshold. Consistent with the Pilot study, the viewing distance had a constant effect on the threshold (F (2,70) = 50.2708, *p* < 0,0001); however, no significant interaction was revealed between the group and the orientation of the targets. The average contrast detection threshold did not statistically differ between the control and the heterophoric group (F (1,14) = 0.4345, *p* = 0.5205). We also found, using a post-hoc analysis, that the contrast detection thresholds of a single target obtained at 40 cm for both orientations and groups were statistically higher than at 1 m (*p* < 0.001 for all comparisons (see the “Discussion” section). The contrast detection thresholds were also significantly higher at 40 cm than at 60 cm for the control group (for both orientations) and for the heterophoric group, but only for the horizontal orientation (see the “Discussion” section). No statistical difference was found for the average contrast detection thresholds obtained between 60 cm and one meter, for both groups and orientations. Detailed statistical information can be found in Supplementary Material Table 5.

#### Lateral interactions

The threshold elevation represents the changes in the detection of the target (contrast threshold) induced by the flanking masking Gabor’s patches, relative to the standard (no mask) condition, in log units (see Fig. 1B).

##### Comparison between control and heterophoric population

**Figure 2:**
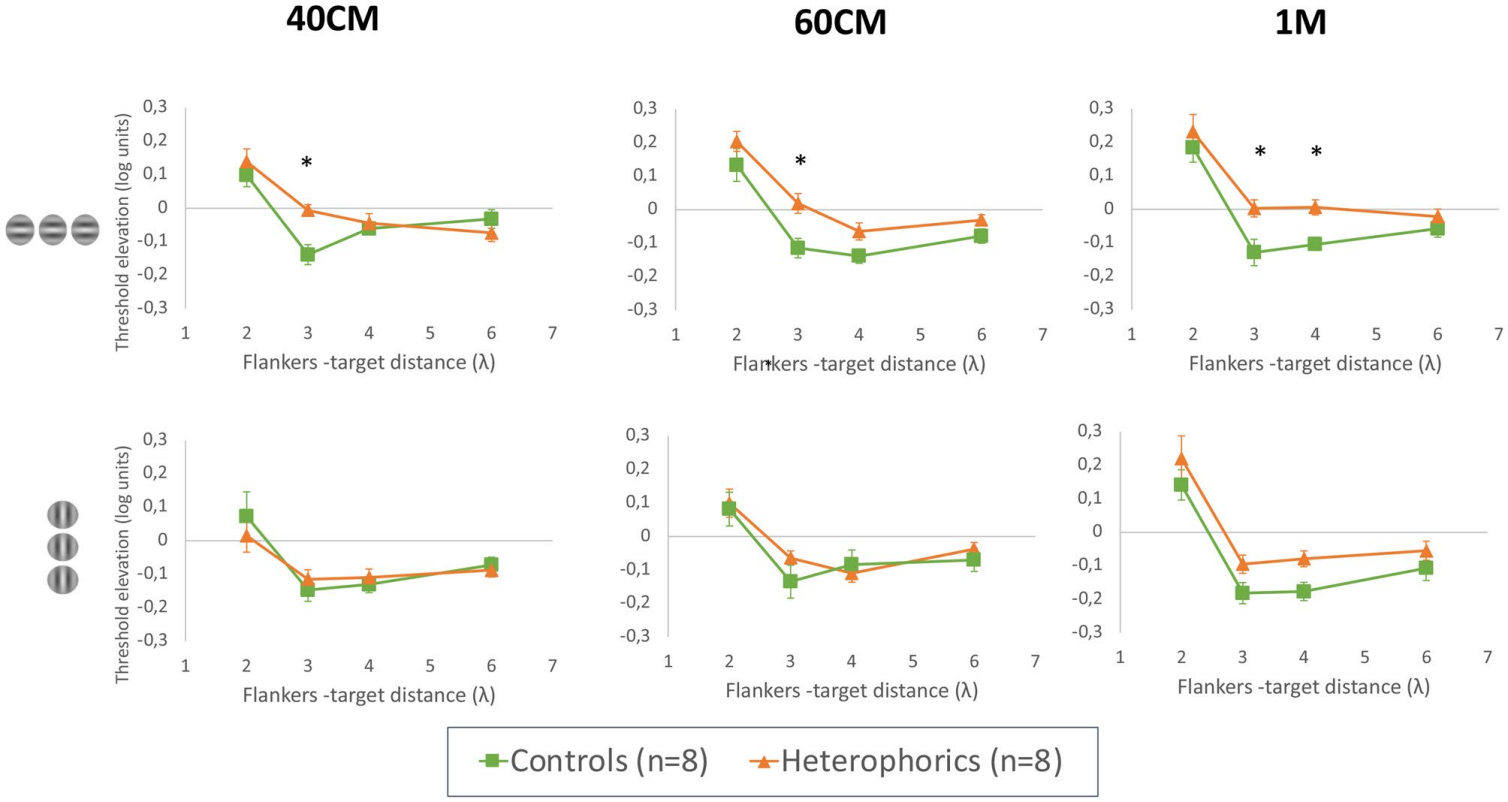
Average threshold elevation for the control group (n = 8) versus the heterophoric group (n = 8) as a function of the flankers to target separation. The control group is denoted by the green line and filled squares; the heterophoric group is denoted by the orange line and the filled triangle. The results for a viewing distance of 40 cm are represented on the left, for 60 cm on the middle, and for one meter on the right. Facilitation is indicated by values below zero, and suppression by values above zero. The upper line of the graphs represents the results for HM and the lower line represents the results for VM. The error bars represent the standard error of the mean (n = 8). ***p ≤ 0.001, **p ≤ 0.01, *p ≤ 0.05.

Regarding the Pilot study (see the Supplementary Material for detailed results), the threshold elevation per subject for each viewing distance was calculated and plotted as a function of the target-to-flanker distance (see Fig. 2). We performed a four-way ANOVA to test the effect of orientation, viewing distance, target-to-flanker separation, and group on threshold elevation. Group (F (1,14) = 6.5397, *p* = 0.0228), viewing distance (F (2,322) = 3.7653, *p* = 0.0242), target-to-flanker separation (F (3,322) = 134.7096, *p* < 0.0001), and orientation (F (1,322) = 26.6293, *p* < 0.0001) had a constant effect on the threshold elevation. There were significant interactions between group and other factors such as viewing distance (F (6,322) = 3.5447, *p* = 0.03) and the flankers’ separation (F (3,322) = 3.7653, *p* = 0.018). There was a significant interaction between viewing distance and target-to-flanker separation as well (F (6,322) = 3.3029, *p* = 0.0036). A post-hoc analysis revealed that subjects with a high phoria showed a significantly higher threshold elevation (less facilitation) than the controls for the target-to-flanker separation of 3λ at all viewing distances. Instead of reaching a minimal threshold elevation (higher facilitation) at 3λ like the controls or the findings in the literature, the phoric group presented an average threshold elevation approaching zero (viewing distance: mean ± SE; 40 cm: -0.006 ± 0.016; 60 cm: 0.019 ± 0.030; 1 m: 0.002 ± 0.025) at all viewing distances, meaning that there was neither facilitation nor suppression at this target-to flanker separation. Nevertheless, no difference was observed between the control group and the phoric group at 2λ, 4λ, or at 6λ. No difference was observed between the two groups for the vertical condition for each viewing distance.

##### Asymmetry of the lateral interactions

**Figure 3:**
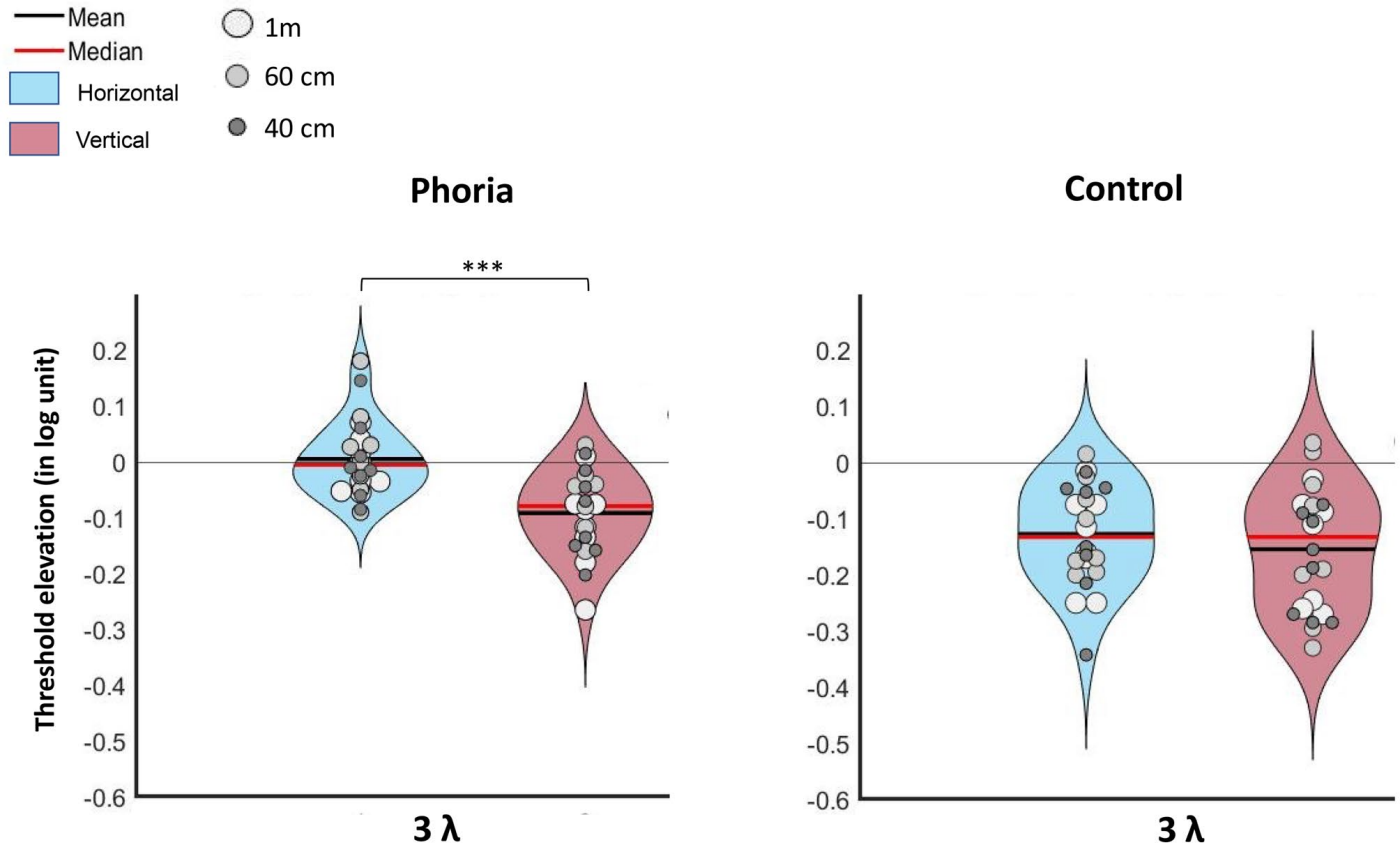
Violin plots represent the distribution of the threshold elevation (in log units) for a target separation of 3λ for the horizontal meridian (the orientation of a target of 0°, in blue) and the vertical meridian (the orientation of a target of 90°, in red). Facilitation is indicated by values below zero, and suppression by values above zero. The results were obtained in experiment 1. The threshold elevations represented were averaged for all the distances. The graph on the left represents the results obtained for the heterophoric group (n = 8), and the graph on the right represents the control group (n = 8). Big-sized circles in light gray denote the threshold elevation for each participant at 1-m viewing distance; middle-sized circles in medium gray denote the threshold elevations for each participant at 60 cm viewing distance, and the little circles in dark gray denote the threshold elevations for each participant at 40 cm viewing distance ***p ≤ 0.001, **p ≤ 0.01, *p ≤ 0.05.

Regarding the Pilot study (see Supplementary Material Pilot study for detailed results for detailed results), we noticed the absence of facilitation or suppression at 3λ for the heterophoria group only on the horizontal meridian. We decided to compare threshold elevation obtained at 3λ for the horizontal meridian and the vertical meridian to determine whether there was also an asymmetry of lateral interaction at this target-to-flanker separation distance. The results are summarized in Fig. 3. A three-way ANOVA was performed to test the effect of viewing distance, group, and orientation on threshold elevation. In this experiment, viewing distance did not have an interacting effect on other factors; thus, the final model included only a significant interaction between group and orientation (F(1,76) = 4.545, *p* = 0.0362). Furthermore, viewing distance did not have a significant effect (F(2,76) = 1.281, *p* = 0.282). The post-hoc analysis revealed that when averaging over viewing distances, there was a significant difference between the horizontal and vertical meridians in the heterophoria group (a mean difference of 0.0967, *p* < 0.0001) but no significant difference in the control group (a mean difference of 0.027, *p* = 0.2451). This highlights again the asymmetry of the lateral interactions for subjects presenting a high heterophoria (see Fig. 3). Since the heterophoria greatly varied between distance and near vision, it was interesting to note that we did not find any effect of the distance on the threshold elevation except for the vertical condition in the heterophoric group for 2λ between the results obtained at 40 cm and at one meter viewing distance (see the “Discussion” section). The threshold elevation was more positive (more suppression) at 1-m observation.

### Experiment 2: Lateral masking in monocular viewing

The results of Experiment 1 revealed an abnormal pattern of lateral interactions for the horizontal meridian, as well as asymmetry of the lateral interactions in binocular viewing. Because heterophoria is a binocular fusion disorder, we decided to run another set of experiments to determine whether the lateral interactions in monocular viewing are normal; however, the abnormal pattern was present only during binocular viewing, suggesting transient binocular suppression. However, if the abnormal pattern is present in monocular viewing, it may suggest a sustained suppression over the monocular viewing or the effect of abnormal development of the monocular lateral interactions.

#### Participants

Data were collected on sixteen subjects. The eight subjects that constituted the heterophoric group were the same as in experiment 1; seven subjects of the control group participated in experiment 1, and one new control subject was added.

#### Single-target detection task

##### Contrast detection threshold

**Figure 4:**
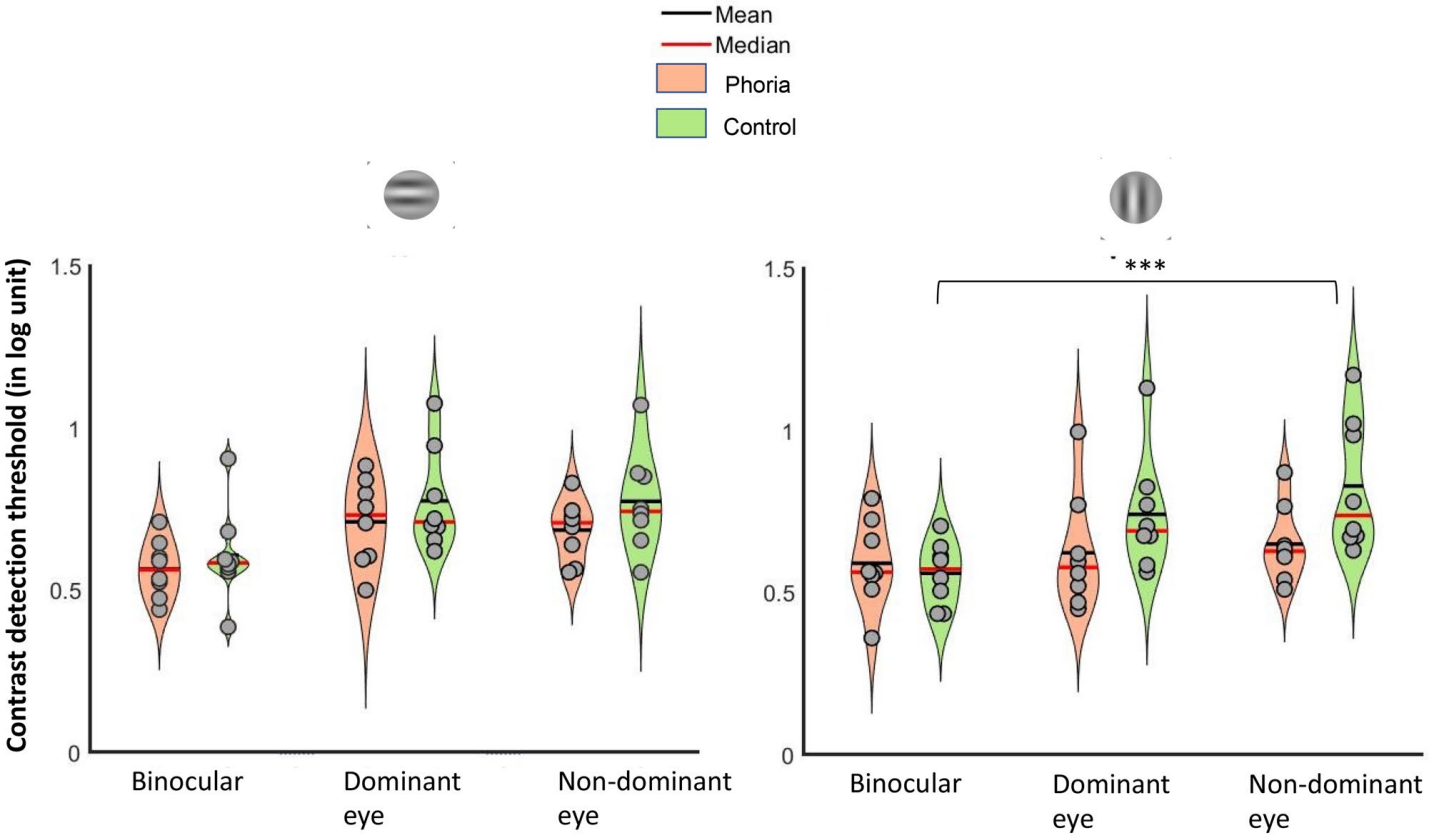
The violin plots represent the distribution of contrast detection threshold for binocular viewing, and both monocular viewings (dominant and non-dominant eye) in Experiment 2 for the control (n = 8) in green and the phoric group (n = 8) in orange for a target orientation of 0° (left) and 90° (right). ***p ≤ 0.001, **p ≤ 0.01, *p ≤ 0.05.

The contrast detection threshold of a single target was measured at 1-m distance under binocular and monocular viewing (both the dominant and non-dominant eye). As shown in Fig. 4, no statistical difference was found between the controls and the heterophoric group for the average contrast threshold detection of the dominant eye (HM: *p* = 0.6785; VM: *p* = 0.3235), non-dominant eye (HM: *p* = 0.5447; VM: *p* = 0.082), and binocular viewing (HM: *p* = 0.7436; VM: *p* = 0.805). We performed a three-way ANOVA to analyze the effect of group, eye viewing (dominant, non-dominant, or binocular viewing) and the target orientation on the contrast detection threshold. The type of eye viewing had a constant effect on the contrast threshold (F (2,70) = 17.2195, *p* < 0.0001). Within the heterophoric group, no significant difference was observed between the different measures for both the vertical and horizontal conditions. Within the control group, a post-hoc analysis revealed that only in the vertical condition was the average threshold for binocular viewing significantly better than for the non-dominant eye (eye viewing: mean ± SE; binocular viewing: 0.57 ± 0.03 log units, non-dominant viewing: 0.87 ± 0.07 log units; *p* = 0.0007), which was in accordance with earlier studies^40,41^.

##### Binocular summation

**Figure 5:**
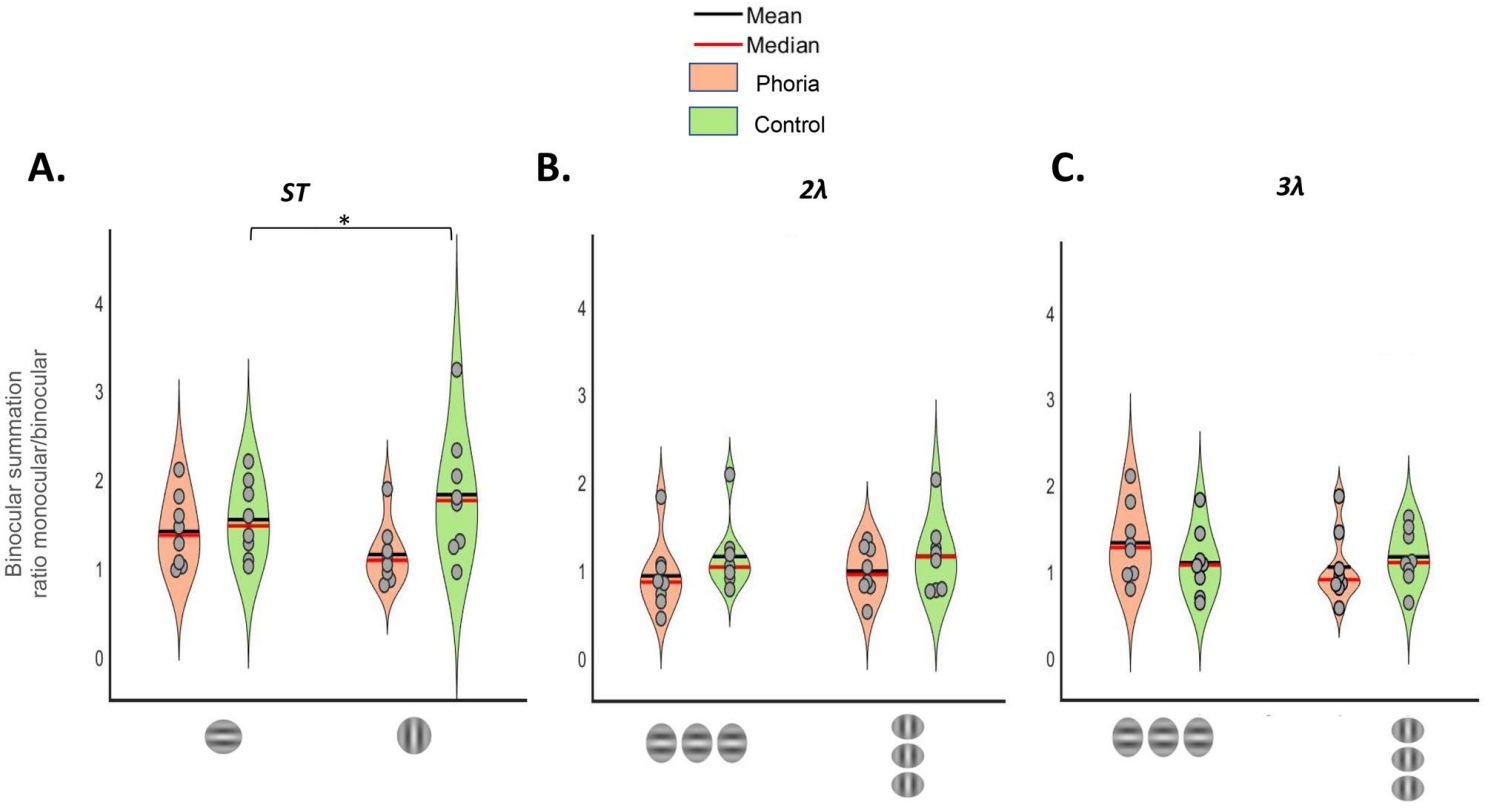
The violin plots represent the distribution of binocular summation for each subject in the heterophoria group (n = 8) in orange and for the control group (n = 8) in green as a function of the target orientation or meridians. (**A**) Binocular summation for the single-target (ST) contrast threshold, expressed as the ratio between the average of the two monocular contrast thresholds and the binocular contrast threshold (in %). (**B**) Binocular summation for the contrast thresholds obtained in lateral masking at 2λ, expressed as the ratio between the average of the two monocular contrast thresholds and the binocular contrast threshold (in %). (**C**) Binocular summation for the contrast thresholds obtained in lateral masking at 3λ. *** *p* ≤ 0.001, **p ≤  0.01, *p ≤ 0.05.

Binocular summation was calculated using the ratio between the contrast thresholds (in %) of the average of the two monocular eyes in relation to the binocular threshold [Monocular average threshold/Binocular threshold]^61,76,77,79^. As seen in Fig. 5A, for the heterophoric groups, we can observe typical binocular summation in the horizontal meridian (1.41 ± 0.14; mean ± SE) but not in the vertical meridian (1.15 ± 0.12; mean ± SE). In the control group, we can observe a binocular summation in the horizontal meridian (1.54 ± 0.15; mean ± SE) and a larger binocular summation in the vertical meridian (1.82 ± 0.25; mean ± SE). We performed a mixed two-way ANOVA to assess the effect of group and orientation on the binocular summation, which showed no significant effect (group: F(1,14) = 3.676, *p* = 0.08; orientation: F(1,14) = 0.2013, *p* = 0.66). We noted a significant interaction of the group and orientation (F(1,14) = 5.052, *p* = 0.04). A post-hoc analysis did not reveal any statistical difference between the two meridians within each group (controls: *p* = 0.22; heterophoria: *p* = 0.15) or between the two groups for the horizontal meridian (*p* = 0.57). For the vertical meridian the binocular summation was significantly lower for the phoria group than for the control group. This can be explained by a lower contrast threshold for a single target for the phoria group for the vertical meridian, as explained above (*p* = 0.05).

#### Lateral interaction

##### Binocular summation

Binocular summation was calculated for contrast thresholds obtained in the lateral masking experiment at 2λ and 3λ using the same method as described above for the single target; the results are summarized in Fig. 5B,C, respectively. We performed a two-way ANOVA to analyze the effect of group and orientation on the binocular summation. At 3λ, both group (F(1,14) = 0.0433, *p* = 0.83) and orientation (F(1,14) = 1.7604, *p* = 0.20) did not have a significant effect. However, there was a significant interaction of the group and orientation (F(1,14) = 6.1043, *p* = 0.03). Similar to the binocular summation obtained for a single target in the heterophoric group, we noted a binocular summation for the horizontal meridian (1.33 ± 0.16; mean ± SE) but not for the vertical meridian (1.05 ± 0.15); it was not statistically different according to the post-hoc analysis (*p* = 0.07). For the control group, there is no binocular summation for both meridians (horizontal: 1.10 ± 0.13; vertical 1.16 ± 0.11; meridian: mean ± SE). The post hoc analysis did not reveal any statistical difference between the two meridians for the control group (*p* = 0.45), or between the two groups for each meridian (horizontal: *p* = 0.45; vertical *p* = 0.45).

Interestingly, the phoria group showed a slight binocular suppression at 2λ for each meridian and especially for the horizontal one (horizontal:0.93 ± 0.15; vertical 0.98 ± 0.10; meridian: mean ± SE). The control group did not show any binocular summation for each meridian (horizontal:1.16 ± 0.14; vertical 1.16 ± 0.15; meridian: mean ± SE). A two-way ANOVA was performed to analyze the effect of group and orientation and did not find any statistical significance (group: (F(1,28) = 2.0392, *p* = 0.1644); orientation (F(1,28) = 0.0448, *p* = 0.8338), nor a significant interaction between group and orientation (F(1,28) = 0.0358, *p* = 0.8514). These results are in accordance with recent findings that detection of the target is more suppressed under binocular than monocular viewing for short target-to-flanker separations^77^.

##### Lateral interactions and the binocular advantage

**Figure 6:**
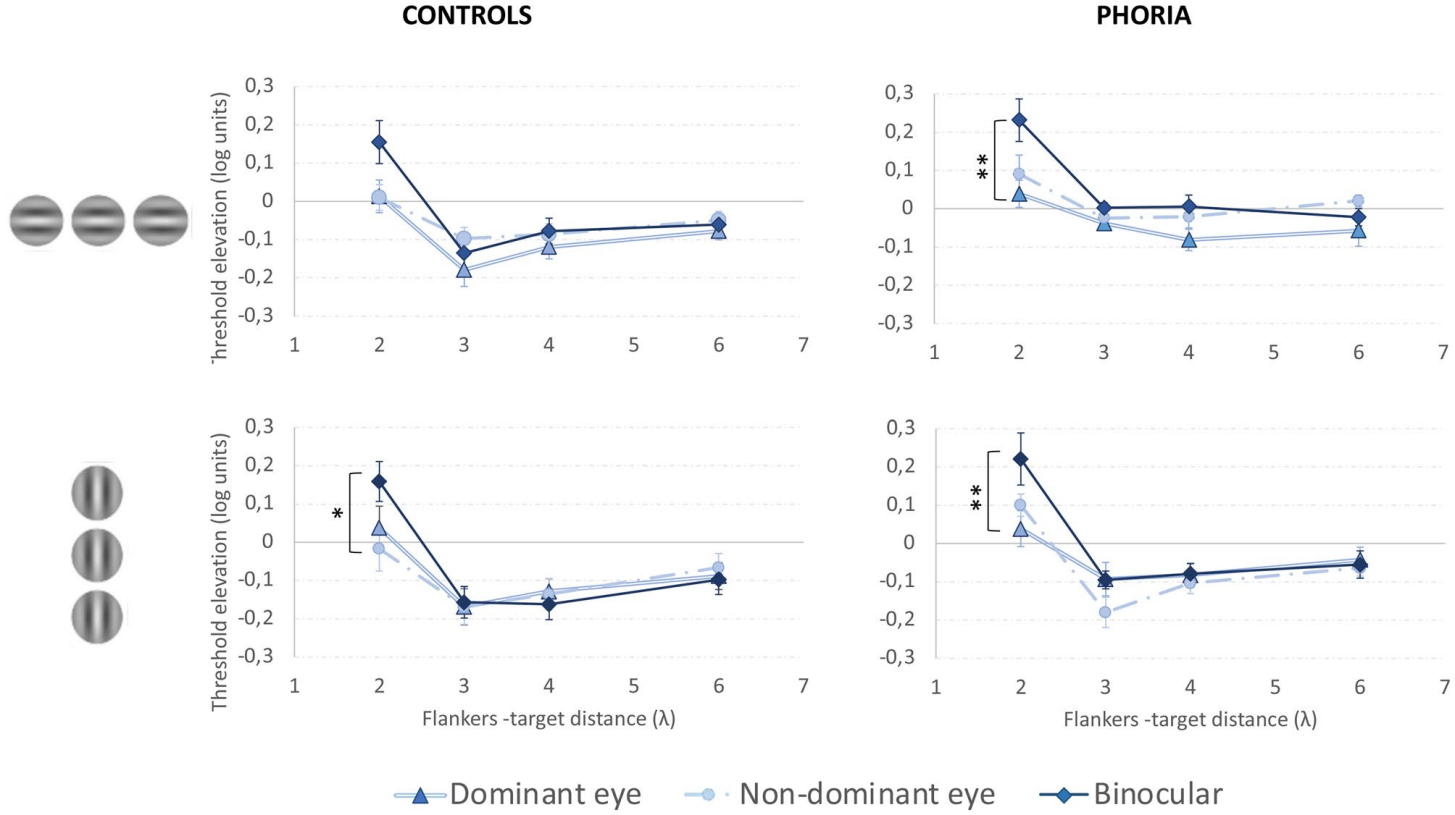
Threshold elevation (in log units) as a function of the flanker-target distance (in λ) for the control group (left) and the heterophoric group (right) at a viewing distance of one meter for a target orientation of 0° (the upper line of graphs) and 90° (the lower line of graphs). Facilitation is indicated by values below zero, and suppression by values above zero. The light blue line and the filled circles denote the results of the non-dominant eye, the double blue line and the filled triangle denote the results of the dominant eye, and the dark blue line with the filled diamonds denotes the results for binocular viewing. The error bars denote the standard error of the mean. ***p ≤ 0.001, **p ≤ 0.01, *p ≤ 0.05.

Threshold elevations obtained for each eye viewing condition (binocular, dominant, and non-dominant eye) and for a target orientation of 0° and 90° were plotted as a function of the flanker-target distances. Figure 6 summarizes the results obtained for this experiment. For the facilitation range, no binocular advantage was found in both groups and orientations, particularly for short target-to-flanker separations, as previously shown in the literature^76,77^. In general, the results obtained when subjects used both eyes together even showed a binocular suppression (i.e., the threshold elevations obtained in binocular viewing were higher than those obtained with both monocular viewings). We performed a four-way ANOVA to assess the effect of group, eye viewing, orientation, and target-to-flanker separations on the elevation threshold. There was a constant effect of all the factors on the elevation threshold: group (F(1,28.0814) = 14.0796, *p* = 0.0008), eye viewing (F(2,322.5311) = 9.8693, *p* = 0.0001), target-to-flanker separation (F(3,322.5311) = 9.8693, *p* < 0.0001), and collinear orientation (F(1,322.5311) = 11.3516, *p* = 0.0008). There was a significant interaction between the target-to-flanker separation and the eye viewing (F (6,322.5311) = 5.4766, *p* < 0.0001). For the heterophoric group, a post-hoc analysis found a statistically greater suppression at 2λ in binocular viewing than with the dominant eye both for vertical (eye viewing: mean ± SE; binocular viewing: 0.22 ± 0.07 log units, dominant eye viewing: 0.04 ± 0.05 log units; *p* = 0.0092) and horizontal conditions (eye viewing: mean ± SE; binocular viewing: 0.23 ± 0.05 log units, dominant eye viewing: 0.04 ± 0.03 log units; *p* = 0.0049). For the control group, the post-hoc analysis showed that the suppression was greater in binocular viewing than with the non-dominant eye at 2λ but only when the Gabor patches were vertically oriented (eye viewing: mean ± SE; binocular viewing: 0.15 ± 0.05 log units, non-dominant eye viewing: −0.02 ± 0.05 log units; *p* = 0.0139).

We did not find any difference in the threshold elevation obtained with binocular viewing and with the dominant and non-dominant eye at 3λ, 4λ, and 6λ, neither in the phoric group nor in the control group in both the horizontal and vertical meridians. Interestingly, in the phoric group, the threshold elevations obtained during monocular viewing seemed to follow the trend of binocular viewing: very poor facilitation at 3λ and normalization of the value of facilitation at 4λ and 6λ. The post-hoc analysis revealed that the threshold elevation of the dominant eye was significantly higher in the heterophoric group than in the control group at 3λ (group: mean ± SE; heterophoria: − 0.04 ± 0.01 log units, control: − 0.18 ± 0.03 log units; *p* = 0.0491), which corroborated the abnormal pattern of collinear facilitation found in the phoric group during monocular viewing. In the control group, threshold elevations obtained by dominant eye viewing were all lower than by binocular viewing through all the target to flanker separations (better facilitation at 3λ, 4λ, and 6λ and no suppression at 2λ); however, threshold elevation was higher at 3λ with the non-dominant eye than with the dominant eye or with binocular viewing.

### Experiment 3: Lateral masking with eye tracker monitoring

One possible explanation for the abnormal pattern of the lateral interactions obtained in the heterophoric group was due to unstable eye fixation during the testing. To verify whether the effect obtained in the heterophoric group was due to a misalignment of the eyes that became manifest (i.e., if an eye turned inward or outward during the experiment), we decided to conduct a control experiment to monitor fixation during the lateral masking (LM) experiment in binocular viewing. The experiment was conducted at 1-m distance, and we tested single-target detection and LM with a target-to-flanker separation of 3λ along the horizontal meridian (where the suppression or absence of facilitation obtained in the LM experiment was more pronounced).

Data were collected on ten subjects (five subjects with high heterophoria and five subjects as the control group). The target (both during the single target detection or during LM at 3λ) was presented either at 300 ms or at 800 ms.

**Figure 7:**
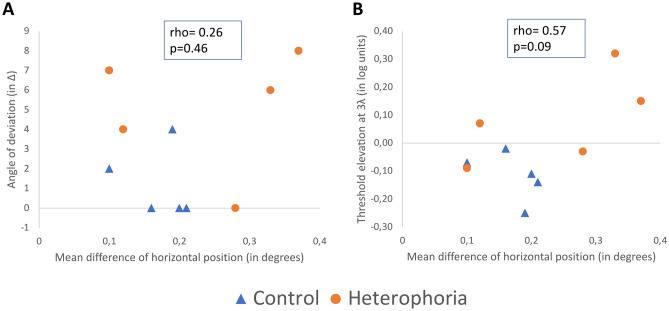
(**A**). Correlation between the angle of deviation (in Δ) and the mean difference in the horizontal position between the two eyes (in degrees). (**B**) Correlation between the threshold elevation at 3λ (in log units) and the mean difference in the horizontal position between the two eyes (in degrees). In both graphs, the blue triangle denotes controls and the orange circle denotes the heterophoric group.

For each control and heterophoric subject, the difference in the mean horizontal position (including the standard error of the mean) between the two eyes was less than 0.5° (group: mean ± SE; controls: 0.17 ± 0,02°; heterophoria: 0,24 ± 0,06°). This range corresponds to the size of the fovea and the error range of the eye tracker, meaning that both heterophoric subjects and control subjects had sufficiently good fixation during the experiment. At this distance, the size of a 4 cycles per degree (cpd) Gabor is about 0.5°. An independent samples *t*-test was performed and no significant difference in the mean horizontal position between the control and the phoric group was observed (unpaired *t*-test, two-tailed, *p* = 0.352). It is interesting to note that the threshold elevation at 3λ differed significantly between the controls and the heterophoric group (control: − 0.12 ± 0.04; heterophoria: 0.08 ± 0.07; mean ± SE, *p* = 0.05, unpaired two-tailed *t* test). We tested the relationships between the amount of deviation and the mean horizontal position difference. A low correlation (Fig. 7A) was found (Pearson’s correlation, rho = 0.26), without statistical significance (*p* = 0.46). Between the threshold elevation at 3λ and the mean horizontal position difference (Fig. 7B), the correlation was stronger (Pearson’s correlation rho = 0.57); however, it was not yet significant (*p* = 0.08). Thus, apparently some relationship exists between the threshold elevation and the amount of the mean horizontal position difference, but it is still not significant, possibly due to the insufficient sample size.

## Discussion

Heterophoria (phoria) is a latent misalignment of the eyes; it is revealed in conditions that disrupt binocular vision (a prevalence up to 30%), and it affects the quality of binocular fusion. We hypothesize that phoria may affect lateral interactions on the horizontal meridian, due to the latent eye misalignment and its influence on binocular fusion. We investigated how phoria affects lateral interactions during binocular and monocular viewing. Heterophoric observers showed an abnormal pattern of collinear facilitation only for the horizontal meridian for both binocular and monocular viewings: the absence of facilitation or suppression rather than facilitation at 3λ is reminiscent of the lateral interactions for amblyopic subjects. In addition, we observed an asymmetry of lateral interactions (for the vertical meridian, lateral interactions for the heterophoric group were not different from the controls). The abnormal pattern observed at the monocular level suggests a developmental disorder in the inter-ocular balance.

The oculomotor system is not yet mature at birth, but it develops rapidly in parallel with the anatomic development of the eye vision and the visual pathway (sensory system)^94^. At an early age, infants are very sensitive to conditions such as common vision problems (e.g., uncorrected refractive errors, strabismus, and visual deprivation) that interfere with visual development. When vision problems are present during the sensitive period, these conditions may cause a permanent reduction of stereoacuity by blocking the development of stereopsis^95–97^. It has been found that most newborn infants do not display strabismus^98^, and that many of even the youngest infants showed good ocular alignment, both monocularly and binocularly, thus indicating that cortical development rather than ocular alignment is the limiting factor regarding early binocular functions. This suggests that oculomotor constraints are not a significant barrier to the development of higher forms of binocularity^99^.

### Abnormal patterns of lateral interactions in heterophoria: pseudo meridional amblyopic behavior

Phoric observers exhibited several deviations from the normal pattern only for the horizontal meridian during both binocular and monocular viewing.

Amblyopia is characterized by a large range of behavioral, neural, perceptual, oculomotor, and clinical abnormalities (for reviews, see^100,101^). Among them, an abnormal pattern of lateral interactions was observed in functional strabismic amblyopia^85,102,103^: facilitation at 3λ was either markedly lower or replaced by inhibition. We obtained similar results only along the horizontal meridian for the heterophoric group: there was suppression at 3λ, contrary to the normal findings in the literature^40^ with a normal effect around 6λ. The facilitation for the collinear configurations was either lower than normal or replaced by inhibition, reminiscent of the findings in the amblyopic population^102^, suggesting that heterophoria would behave like meridional amblyopia along the horizontal meridian. Such a phenomenon was recently observed; it took place in oblique astigmatism where a decrease in facilitation by 13% was observed for a target oriented similarly to the blurriest meridian^76^.

Note that the abnormal LM in amblyopia is present only in the amblyopic eye, whereas the results in the fellow eye are normal^85,102^. Thus, we wanted to investigate whether this pattern is restricted to binocular viewing, when subjects display high heterophoria. If the effect described here resulted from poor binocular fusion due to an oculomotor disorder during the experiment with binocular viewing (i.e., intermittent or manifest misalignment of eyes), we would have expected to observe normalization of the threshold elevations during monocular viewing. However, within the phoric group, for both the dominant and non-dominant eyes, the threshold elevations seemed to follow the pattern of the threshold elevations obtained with binocular viewing: minimal or no facilitation at 3λ. Moreover, statistically less facilitation was found with the dominant eye in the phoric group than in the control group, reminiscent of the monocular abnormal lateral interaction in amblyopia, showing an increased range of suppression. This effect can be explained as a developmental disorder of the neural interaction in the amblyopic eye, whereas the lateral interaction in the fellow eye is normal^85,102^. Here, we found that the lateral interactions in both eyes for the heterophoric group are abnormal. The difference between these two conditions could be explained by the fact that in amblyopia there is a strong difference between the two eyes; thus, one eye suppresses the other^70,104^, whereas in the heterophoric group, the two eyes are almost equal. Our results as well as the eye tracker recordings stand in favor of a developmental cortical disorder similar to meridional amblyopia, regardless of the refractive error (no high astigmatism in the inclusion criteria).

### Asymmetry of lateral interactions in heterophoria

We noticed an abnormal pattern of lateral interaction for the horizontal meridian in the phoric group, and we also observed for each heterophoric subject that the expected facilitation at 3λ was maintained along the vertical meridian. This asymmetry of lateral interactions was also apparent during monocular viewing: suppression or absence of facilitation at 3λ for both eyes at the horizontal meridian but facilitation at 3λ at the vertical meridian. It was suggested that the suppressive zone is indicative of the perceptive field size (the perceptive field is the psychophysical analog to the classical receptive field in the visual cortex, for a review, see^48,105,106^). Recently, it was shown that the binocular perceptive field size decreases with development, reaching an adult’s size in up to 5–6 years^59^; however, these findings did not determine whether the perceptive field size would be different between the horizontal and the vertical meridian in general, and in heterophoria, in particular.

### Contrast threshold detection of a single target

We found higher contrast thresholds, for the same spatial frequencies, for both groups and for the two meridians when the stimulus was presented at a viewing distance of 40 cm and 60 cm. We suggest that two possibilities could explain the higher contrast thresholds. The first could be accommodation; we know that accommodation capacity decreases with age (presbyopia) and that participants at 40 cm should perform an accommodation of 2.5 diopters. The second can be due to the lower resolution of the monitor for the target at 40 cm. To enable the experiment to be completed in the same experimental condition at each distance, the size of the Gabor patch must be adjusted when it is projected at one meter or 40 cm. Considering this, one cycle (λ) of a 4-cycle per degree (cpd) of a Gabor patch is represented by 16 pixels on the monitor screen for one meter, whereas at 40 cm it is represented by 6 pixels. Note that for our Gabor patch (λ = σ) there are at least 2 cycles per Gabor patch. The overall resolution of the target is therefore lower at 40 cm; this could explain the higher contrast detection thresholds obtained. We believe that this last explanation is the most probable: first, previous studies showed better visual acuity for distance^107^; second, the enrolled participants are young enough in both groups not to be presbyopic (a maximum age of 35 years old) and they do not show any accommodative disorders either, since these disorders were part of our exclusion criteria.

### Binocular summation

#### Single-target contrast detection

In the heterophoric group, the binocular summation is typical for the horizontal meridian but not for the vertical meridian, which shows no binocular summation. Indeed, the binocular contrast thresholds of a single target in the vertical meridian were almost equal to those of the monocular thresholds. One reason could be that the two eyes do not fully align (a small phase shift between the eyes) on the vertical Gabor, which creates a difference in the phase shift in each eye during binocular viewing, impairing the binocular summation^70,108,109^. An alternative possibility of depth perception is less likely, due to the short presentation time (80 ms) that was used here and from testing performed in the pilot experiments. Moreover, none of our participants reported seeing any depth.

#### Lateral interactions

Both groups did not show any binocular summation at 2λ; for the heterophoric group, there was even a slight binocular suppression, however not significant. The binocular summation for 3λ generally behaved like the binocular summation found for the single target. The absence of binocular summation at 2λ is in accordance with previous studies that found no summation for short target-flanker distances, suggesting a combination of interocular suppression with an additional suppression from lateral interaction from inside the perceptive field^77^. We found a greater suppression at 2λ for binocular viewing for each group, in accordance with the findings in the literature^77^. During the lateral masking experiments, for both groups and both meridians, threshold elevations were higher at 2λ with binocular viewing than with monocular viewing and were almost the same from the target-to-flanker separation of 3λ. Nevertheless, for the heterophoric group, threshold elevations under binocular viewing were systematically higher for all target-to-flanker distances along the horizontal meridian, contrary to recent findings in the literature, where the binocular advantage is restored at larger target-to-flanker distances^77^. An explanation for this effect could be a greater interocular suppression in heterophoria along the horizontal meridian. In addition, it is interesting to note that in both groups, threshold elevations obtained for both the horizontal and vertical meridians were not statistically lower (no improvement in the facilitation) during binocular viewing than during monocular viewing, reinforcing recent findings that two eyes are not better than one with cases of contextual modulations^76,77,79^.

### Distance effect

The amplitude of heterophoria varies with the distance of the observation. In our sampled population and in general, horizontal heterophoria was mainly present at a near distance. We did not observe any statistical effect of the distance, although the amplitude of phoria was almost double at 40 cm. A limitation or the absence of facilitation at 3λ in the horizontal meridian was even more pronounced at 1-m distance. Even if the initial contrast threshold for the single target at 1-m distance, which was statistically similarly low for both groups, could explain the limitation for greater facilitation at 3λ, there was still a significantly greater facilitation at 3λ for the controls than for the heterophoric group. In other words, when subjects present high phoria, even if their phoria is high only at a near distance and not a 1-m distance, they will show very limited facilitation or suppression at 3λ in the horizontal meridian regardless of the distance at which they observed the stimuli. This observation supports the hypothesis that high horizontal heterophoria could reflect a developmental impairment in the visual system.

### Implications in daily activities

It was found that for horizontal heterophoria, the binocular advantage experienced during reading (a shorter fixation duration or shorter sentence reading times) is reduced and for large heterophoria, binocular reading turned even into a disadvantage^17^. Since reading occurs horizontally, we suggest that the abnormal pattern found for the horizontal meridian only in the phoria group as well as the absence of a binocular advantage for the facilitation could contribute to explaining the slower sentence reading and the longer fixation times for subjects presenting heterophoria. Regarding the computer vision syndrome (CVS), because of prolonged reading in front of screens, and the relatively high prevalence of heterophoria (up to 35%), we suggest that our results might well explain, at least in part, the visual symptoms encountered in populations suffering from this syndrome. Few studies have investigated whether heterophoria changes after prolonged use of head-mounted devices (HMDs) for virtual reality (VR) use and contradictory findings were observed. Some researchers found that heterophoria remained the same before and after the use of HMDs^2^, whereas others found an esophoric shift^110^, or that participants with a large exophoria were more prone to worsening of exophoria than those with a small exophoria^111^. Nevertheless, no studies investigated whether the visual discomfort or other symptoms reported with the use of HMDs were correlated with the presence of heterophoria. We proposed, since heterophoria seems to affect visual processes implicated in contour detection and grouping, that the presence of heterophoria might contribute to experiencing visual symptoms during the use of HMDs for VR.

## Conclusion

Heterophoria affects spatial interactions in a way that is reminiscent of meridional amblyopia without being attributed to abnormal refraction^102^. The existence of abnormal collinear facilitation in monocular viewing suggests that phoria could be a binocular developmental disorder that affects monocular spatial interactions. No binocular advantage of the threshold elevations was found, confirming recent findings that sometimes two eyes are not better than one in cases of contextual modulations^76,77,79^. In light of our results, and since (1) both crowding and masking are dependent on the perceptual field size^48,59,102,112^ and (2) crowding is similar to lateral interactions in certain spatial and temporal conditions^48^, we suggest that heterophoria could affect the symmetry of the perceptive field as well as the crowding effect—a hypothesis that deserves further investigation.

Although heterophoria is not necessarily pathological, this condition is frequently encountered by vision specialists and is often associated with the increasingly prevalent computer vision syndrome (CVS). We suggest that our findings could explain CVS-associated symptoms as well as other phenomena linked to the use of head-mounted visual display (HMD) of virtual reality (VR) such as visual discomfort and fatigue^2^ or the lack of binocular advantages found in reading subjects that present high heterophoria^17^.

To end on a methodological note, we need to remember that heterophoric subjects present normal visual acuity and normal stereoscopic vision. Therefore, our findings highlight the importance of taking into consideration heterophoria as a criterion of exclusion (or inclusion) for studies that investigate visual processing.

## Supplementary Information

Supplementary Information.
