# Supplementary material for: Binocular fusion disorders impair basic visual processing
Source: Sci Rep. 2022 Jul 22;12:12564. doi: 10.1038/s41598-022-16458-y (PMC9307628; doi:10.1038/s41598-022-16458-y)
Supplement: Supplementary file 1 — Supplementary Information. [file 41598_2022_16458_MOESM1_ESM.docx]

**Binocular fusion disorders impair basic visual processing**

Laura Benhaim-Sitbon, Maria Lev, and Uri Polat

School of Optometry and Vision Sciences, Faculty of Life Sciences, Bar-Ilan University, Ramat-Gan, Israel

## **Supplementary Material**

## Heterophoria

In everyday life, when looking at an object, each eye receives a slightly different image due to its anatomical position on the head. Binocular vision refers to a condition where the two eyes view a common portion of the visual space, up to about 190° in humans^1^ . Normal binocular vision enables one to apprehend slightly dissimilar inputs as a single image and requires proper alignment of the two eyes, more precisely, proper alignment of the two foveae with the object of interest. This alignment is controlled by sensory and motor mechanisms, both working as a balance system; they are responsible for the binocular fusion of the images.

The motor fusion mechanism is triggered by retinal disparity (slightly different images), so that corresponding retino-cortical elements between the two eyes (retinal elements of the two eyes that share a common subjective visual direction are called corresponding retinal points) are placed in a position to deal with the two images^2,3^. The sensory fusion mechanism is triggered by retinal blur; to occur, images must be located on corresponding retinal areas and must be sufficiently similar in size, brightness, and sharpness^3–5^.

Worth ^6^ classified binocular function into three hierarchically connected levels: bifoveal fixation, fusion, and stereopsis: in normal adults, the presence of bifoveal fixation is necessary for fusion to occur, and fusion is essential for stereopsis. These binocular functions are not mature at birth and develop during the first months of life^7,8^.

If sensory fusion is artificially suspended (by covering one eye, for example), motor fusion is perturbed^9^ and a measurable relative deviation of the visual axes will appear in most individuals. When removing the obstacle to sensory fusion, motor fusion should bring back the visual axes to their correct relative positions. These deviations, after being provoked, are called heterophorias.

The prevalence of heterophoria is better documented in children and in young adults (pre-presbyopic); it varies greatly between studies, mainly because of the use of different testing procedures and diagnostic criteria^10^. Thus, the prevalence of heterophoria in these populations can range from 9.9% to 61.4% (median prevalence: 22.6%)^11^. Phoria obtained at near viewing is overall more prevalent than at distant viewing^11^. For horizontal heterophoria, exophoria (outwards deviation) is more prevalent than esophoria (inwards deviation) in all age groups^12,13^. Heterophoria also depends on refraction and age^13–19^.

Based on clinically significant findings, a certain amount of heterophoria is considered to be normal (see Appendix Table 1). Quantification of the amount of heterophoria (the angle of the decompensated eye misalignment) is usually expressed as a prismatic diopter, a unit specifying the amount of light deviation by an ophthalmic prism. When a light beam goes through a prism, it deviates according to its power. One prism diopter (Δ) corresponds to a deviation of 1cm at 1m.

Table 1: Physiological ranges for heterophoria in the literature.

| **Source** | **Horizontal phoria** | | **Vertical phoria** |
| --- | --- | --- | --- |
|  | **Exophoria** | **Esophoria** |  |
| Scheiman and Wick^5^ | Distance: 1Δ ±2Δ  Near: 3Δ ±3Δ | - | - |
| Moses^20^ | up to 4Δ | Up to 2Δ | 0.5Δ |
| Hashemi et al^11^ | Distance : 3Δ  Near: 9Δ | 1Δ | - |
| Guide for the aviation medical examiner of the Federal Aviation Administration^21^ | 6Δ | 6Δ | 1Δ |

##

## Measurement of heterophoria - procedures

In order to reveal the full deviation, all peripheral visual inputs must be suppressed. Occlusion with an opaque occluder held close to the eyes was used to achieve maximal dissociation. All measurements were realized in the primary viewing position. Measurements of both the horizontal and vertical deviations were performed for each subject at each viewing distance of the experiment using the Maddox rod test (MRT) and the alternating cover test (CT). For detailed procedures, see the following two test procedures:

#### Maddox rod test (MRT)

The Maddox rod test (MRT) is a subjective measurement of the presence and extent of ocular deviation^22^. The Maddox rod is a handheld instrument composed of a red parallel plano-convex cylinder lens.  It refracts light rays so that a point source of light is seen as a line or streak of light. The line appears to be perpendicular to the axis of the cylinder.  The superimposition of the Maddox on one eye when both eyes are looking to a light source creates the dissociation of the fusion. The subject is told to look at a light source at 4 m, 1m, and 60cm (only for experiment 1), and at 40cm. To measure a horizontal deviation, the Maddox rod is placed on the non-dominant eye with its cylinder positioned horizontally. A prism bar is applied on the Maddox rod until the red line is perceived on the light source. Because the prism bar has steps of 2 Δ, if, for example, the red line is perceived on the left side of light with a prism of 2Δ and on the right side when moving the prism bar to the next prism (4Δ), the value of 3Δ for the angle of deviation is attributed to it. To measure the vertical deviation, the Maddox rod is placed with its cylinder positioned vertically and the same procedure is repeated.

#### Cover/ uncover test

The cover test or cover/ uncover test is an objective measurement of the presence and extent of ocular deviation. It can assess both heterophoria and heterotropia.

We used an opaque standard-sized occluder and the prism neutralized ACT (Alternating Cover Test) procedure ^23^. This procedure was repeated with a fixation letter of 20/30 placed at 4m on the ETDRS chart, 1m (projected on the monitor screen); 60cm is relevant to the viewing distance of the experiment (also with a letter E projected on the monitor screen) and 40cm using the letter E of the Lang fixation cube (Haag Streit UK Ltd.). We used an accommodative target to mimic as close as possible the visual conditions of the experiment (Gabor patch elicit as well as accommodation). At each distance, the subjects were instructed to fixate on the target and to “keep it clear”, in full room illumination, when they were wearing their glasses or contact lenses.  First, the opaque occluder is placed in front of the left eye, and movement of the right eye is observed; the procedure was repeated with the occluder in front of the right eye. In the absence of movement of the unoccluded eye, heterotropia is excluded.

Then, the occluder is moved alternatively in front of the left and the right eye, and the movement of the right eye is observed. The prism bar is placed no farther than 1 cm from the right eye while performing the alternating cover test (interposing the prism bar behind the occluder).  The prism power is increased until the first reversal (for example, the subject was initially ‘exo’ and now becomes ‘eso’ through the prism). The amount of prism that results in neutrality before this reversal of movement is recorded as the high neutral value.

## Pilot study

#### Participants

Twenty-six subjects participated. The subjects were divided into two groups: one group of 13 subjects with significant heterophoria (≥ 6Δ) for at least one testing distance including 4 participants with esophoria and 9 participants with exophoria as well as one group of 13 subjects with no or negligible phoria (< 6Δ) at all testing distances. No clinically significant vertical phoria was recorded in either group. The age of the subjects was between 19 and 35 years old (27.5± 5.10 years, mean± STD). In the heterophoric group, the age was between 19 and 34 years old (28.46± 5.56 years, mean± STD) and in the control group it was between 20 and 35 years old (26.53± 4.61 years, mean±STD). Each subject in both groups presented a clinically normal stereoscopic vision (40 arcsec).

In the heterophoric group, four subjects presented an esophoria, and nine subjects presented an exophoria. To calculate the power of the horizontal phoria, we referred to the absolute value of the angle deviation regardless of the direction of the deviation. We attributed orthophoria the value 0 Δ, and for an esophoria or an exophoria of *n*Δ , we attributed the same value *n*Δ. For subjects presenting a non-clinically significant vertical phoria (i.e., that was inferior or equal to 1Δ), we attributed only the value for their horizontal phoria.

A three-way ANOVA test was performed to evaluate the effect of group, distance, and methods on the heterophoria measurement. Group (F (1,9) = 16.8281, p=0.0027) and distance (F (1,27) = 6.6, p=0.016) had a constant effect on the measurement obtained. No effect of the methods was observed: the amount of phoria measured by the Cover test (CT) and the Maddox rod test (MRT) was statistically similar. A post-hoc analysis revealed that the amount of phoria was significantly different between the control and the heterophoria group at one meter with both MRT and CT (p<0.05 for all comparisons). Detailed statistical information about measurements, methods, and the amount of phoria in different groups can be found in Appendix Table 2.

#### Contrast detection threshold for a single target

First, we measured the single-target contrast detection threshold at each viewing distance and for two orientations (0° or 90°). The values of contrast detection threshold obtained and other statistical details can be found in Appendix Table 3. For each viewing distance, the average contrast detection threshold was not statistically different between the control and the heterophoric group or between the horizontal and vertical conditions (target orientations of 0° or 90°, respectively) for the heterophoric group. For the control group and only at one-meter distance, the average threshold was significantly higher for a target orientation of 0° than for a target orientation of 90° (p=0.01). A three-way ANOVA was performed to test the effect of global orientation, viewing distance, and group on the contrast detection threshold. There was a significant interaction between group and orientation (F (1,72) =6.5274, p=0.0127), whereas the viewing distance had a constant effect on the threshold (F (1,72) =127.8883, p<0.0001).

Interestingly, a post-hoc analysis revealed that the overall average contrast threshold for a one-meter viewing distance was statistically lower than at 40 cm for both groups (see the *Discussion*).

#### Lateral interactions

For each distance, the threshold elevation per subject was calculated and plotted as a function of target-to-flanker distance. The threshold elevation represents the changes in the detection of the target (contrast threshold) induced by the flanking masking Gabor’s patches, relative to the standard (no mask) condition, in log units (see Figure 1B).

##### Comparison between the control and the heterophoric population

As presented in Figure A1, for the horizontal meridian, facilitation was present, as expected from the literature^24,25^ for the control group and at all viewing distances for each flanker-to-target distance. However, for subjects presenting a clinically significant phoria, no facilitation was found at 40 cm at 3λ (mean±SE; phoric group: -0.013± 0.017 log units) and suppression (a positive value for threshold elevation) was recorded at a one-meter viewing distance. The suppression slowly decreased towards a normal value at 6λ. A four-way ANOVA was performed to test the effect of orientation, viewing distance, target-to-flanker separation, and group on the threshold elevation. There are significant interactions between the group and other factors such as viewing distance (F (1,263.716) =10.1816, p=0.0016), target-to-flanker separation (F(2,263.7153)=7.0339,p=0.0011), and orientation of the collinear Gabor patches (F(1, 263.716)=11.4537, p=0.0008). A post-hoc analysis revealed that the threshold elevation at 3λ was significantly higher for the phoric group than for the control group at one meter ((mean±SE; phoric group: 0.0325 ± 0.034 log units; control group: -0.142 ±0.033 log units, p=0.0022). For the vertical meridian (VM), we obtained, in both groups, facilitation at each target-to-flanker distance, which was in agreement with the findings in the literature^24^. The differences in threshold elevation for 3λ at 40cm and one meter in the heterophoric group can be explained by the higher contrast detection threshold for a single target at 40cm.

Figure S1: Threshold elevation (in log units) as a function of flanker-target distance (in λ units) for the control group (n=13) and the heterophoric group (n=13) at a viewing distance of 40 cm (left) and one meter (right) for a target orientation of 0° (the upper line of the graphs) and 90° (the lower line of the graphs). Facilitation is indicated by values below zero, and suppression by values above zero. The orange line and filled circles denote the heterophoric group; the green line and the filled square represent the control group. The error bars denote the standard error of the mean. *** p<=0.001, **p<=0.01, *p<=0.05

##### Asymmetry of the lateral interactions

Because the absence of facilitation or even suppression at 3λ only in the horizontal meridian for the heterophoric group could evoke an asymmetry of the lateral interactions, we wanted to examine the elevation threshold obtained at 3λ for all meridians and groups. As represented in Figure A2, the threshold elevation at 3λ for the heterophoric group was either close to zero (40cm) or suppressed (1 meter) only for the horizontal meridian, whereas facilitation was present for the vertical meridian. We performed a three-way ANOVA to assess the effect of viewing distance, group, and orientation on threshold elevation. Group (F(1,27.9981)=6.9513, p=0.0135) and orientation (F(1,72.4507)=17.5642, p=0.0001) had a significant effect. In addition, group had a significant interaction with the viewing distance (F(1,72.4507)=4.0594, p=0.0476) and with the orientation (F(1,72.4507=9.6543, p=0.0027). Interestingly, the post-hoc analysis revealed a significant difference in threshold elevation at 3λ between the horizontal and the vertical meridians within the phoria group, at both 40cm ((mean±SE; HM: -0.013± 0,017 log units; VM: -0.136 ±0.018 log units, p=0.0053) and at one-meter viewing distance ((mean±SE; HM: 0.032± 0.034 log units; VC: -0.108 ±0.030 log units, p=0.0015), showing an asymmetry of the collinear facilitation. Generally, for all target-to-flanker separations, the threshold elevation in the horizontal condition was higher than in the vertical condition, thus corroborating the asymmetry of the lateral interactions. We did not find any statistical asymmetry between the horizontal and vertical lateral interaction in the control group, either at 40cm or at one-meter viewing distance throughout the whole experiment (Figure A2).

Figure S2: The violin plots represent the distribution of the threshold elevation (in log units) for a target separation of 3λ for the horizontal meridian (the orientation of a target of 0°, in blue) and the vertical meridian (the orientation of a target of 90°, in red). Facilitation is indicated by values below zero, and suppression by values above zero. Results obtained in the Pilot study: the heterophoria group (n=13) is represented on the left, and the control group (n=13) on the right. The first line of graphs represents the results for a viewing distance of 40cm and the second line represents the results for a viewing distance of one meter. *** p<=0.001, **p<=0.01, *p<=0.05

##### Correlation between the amount of phoria and the suppression at 3λ

We analyzed whether a correlation exists between the amount of phoria (the angle of the phoria) and the threshold elevation obtained at 3λ for a stimulus orientation of 0° at each viewing distance (see Figure S3 ). For a 40 cm viewing distance, a mild correlation was found (Spearman’s rank correlation rho=0.5) but without statistical significance (p=0.07). However, at 1m, the correlation was strong (Spearman’s rank correlation rho=0.7) and significant (p=0.007). The correlation difference between two viewing distances could be explained by the fact that the contrast detection thresholds of the single target are higher at 40cm, leaving more opportunity for facilitation to occur (a decrease in the target contrast detection when the target is flanked by a mask).

Figure S3: Correlation between threshold elevation (log unit) and the absolute value of the angle of deviation (Δ) obtained for a target-to-flanker distance of 3λ, in the horizontal condition at 40cm, a viewing distance (left), and at a one-meter viewing distance (right).

## Statistical information

| Table 2 : Statistical information on the amount of phoria measured by CT (cover test) and MRT (Maddox rod test) for the control and the heterophoric groups enrolled in the Pilot study. In each row, the two parameters in bold represent the two conditions with which the statistical analysis is compared. The evaluation was done using post-hoc analysis of 3-way ANOVA.   \| Method \| Viewing distance (cm) \| Group \| Mean±SE \| P-value \| \| --- \| --- \| --- \| --- \| --- \| \| **CT - MRT** \| 40 \| control \| 4,312±0,804; 4,666±0,840 \| 0.3777 \| \| **CT - MRT** \| 100 \| control \| 2,866±0,589; 3,308±0,710 \| 0.4449 \| \| **CT - MRT** \| 40 \| heterophoria \| 8,615±1,156; 9,153±1,731 \| 0.579 \| \| **CT - MRT** \| 100 \| heterophoria \| 3,923±1,117; 5,077±1,457 \| 0.4449 \| \| CT \| **40 - 100** \| control \| 4,312±0,804; 2,866±0,589 \| 0.1858 \| \| CT \| **40 - 100** \| heterophoria \| 8,615±1,156; 3,923±1,117 \| 0.4449 \| \| MRT \| **40 - 100** \| control \| 4,666±0,840; 3,308±0,710 \| 0.1244 \| \| MRT \| **40 - 100** \| heterophoria \| 9,153±1,731; 5,077±1,457 \| 0.579 \| \| CT \| 40 \| **control - heterophoria** \| 4,312±0,804; 8,615±1,156 \| 0.0551 \| \| CT \| 100 \| **control - heterophoria** \| 2,866±0,589; 3,923±1,117 \| 0.0191 \| \| MRT \| 40 \| **control - heterophoria** \| 4,666±0,840; 9,153±1,731 \| 0.1216 \| \| MRT \| 100 \| **control - heterophoria** \| 3,308±0,710; 5,077±1,457 \| 0.0191 \| |
| --- | --- | --- | --- | --- | --- | --- | --- | --- | --- | --- | --- | --- | --- | --- | --- | --- | --- | --- | --- | --- | --- | --- | --- | --- | --- | --- | --- | --- | --- | --- | --- | --- | --- | --- | --- | --- | --- | --- | --- | --- | --- | --- | --- | --- | --- | --- | --- | --- | --- | --- | --- | --- | --- | --- | --- | --- | --- | --- | --- | --- | --- | --- | --- | --- | --- |

*Table 3: Statistical information on the contrast threshold for each group in the Pilot study. In each row, the two parameters in bold represent the two conditions with which the statistical analysis is compared. The evaluation was done using post-hoc analysis of 3-way ANOVA. H stands for horizontal and V for vertical.*

| Meridians | Viewing distance | Groups | Mean±SE | P-value |
| --- | --- | --- | --- | --- |
| **H - V** | 40 | control | 0,790±0,010; 0,759±0,026 | 0.5275 |
| **H - V** | 100 | control | 0,654±0,042; 0,545±0,026 | 0.0102 |
| **H - V** | 40 | heterophoria | 0,810±0,017; 0,839±0,030 | 0.5275 |
| **H - V** | 100 | heterophoria | 0,574±0,020; 0,594±0,031 | 0.597 |
| H | **40 - 100** | control | 0,790±0,010; 0,654±0,042 | 0.0012 |
| H | **40 - 100** | heterophoria | 0,810±0,017; 0,574±0,020 | >0.0001 |
| V | **40 - 100** | control | 0,759±0,026; 0,545±0,026 | >0.0001 |
| V | **40 - 100** | heterophoria | 0,839±0,030; 0,594±0,031 | >0.0001 |
| H | 40 | **control - heterophoria** | 0,790±0,010; 0,810±0,017 | 0.597 |
| H | 100 | **control - heterophoria** | 0,654±0,042; 0,574±0,020 | 0.0665 |
| V | 40 | **control - heterophoria** | 0,759±0,026; 0,839±0,030 | 0.0665 |
| V | 100 | **control - heterophoria** | 0,545±0,026; 0,594±0,031 | 0.3063 |

Table 4 : Statistical information on the amount of phoria measured by CT (cover test) and MRT (Maddox rod test) for the control and the heterophoric groups enrolled in Experiment 1. In each row, the two parameters in bold represent the two conditions with which the statistical analysis is compared. The evaluation was done using post-hoc analysis of 3-way ANOVA.

| Method | Viewing distance | Group | Mean±SE | P-value |
| --- | --- | --- | --- | --- |
| **CT - MRT** | 40 | control | 2,125±0,671; 2,375±0,770 | 0.6739 |
| **CT - MRT** | 60 | control | 0,750±0,342; 1,000±0,353 | 0.6183 |
| **CT - MRT** | 100 | control | 0,250±0,234; 0,625±0,303 | 1 |
| **CT - MRT** | 40 | heterophoria | 9,000±1,414; 8,250±1,333 | 0.4998 |
| **CT - MRT** | 60 | heterophoria | 4,625±1,084; 4,625±0,865 | 0.9241 |
| **CT - MRT** | 100 | heterophoria | 2,625±0,980; 3,250±0,773 | 0.4138 |
| CT | **40 - 60** | control | 2,125±0,671;0,750±0,342 | 0.2238 |
| CT | **40 - 100** | control | 0,250±0,234; 2,125±0,671 | 0.059 |
| CT | **60 - 100** | control | 0,750±0,342; 2,125±0,671 | 0.8625 |
| CT | **40 - 60** | heterophoria | 9,000±1,414; 4,625±1,084 | 0.0211 |
| CT | **40 - 100** | heterophoria | 9,000±1,414; 2,625±0,980 | 0 |
| CT | **60 - 100** | heterophoria | 4,625±1,084; 2,625±0,980 | 0.0436 |
| MRT | **40 - 60** | control | 2,375±0,770; 1,000±0,353 | 0.26 |
| MRT | **40 - 100** | control | 2,375±0,770; 0,625±0,303 | 0.0203 |
| MRT | **60 - 100** | control | 1,000±0,353; 0,625±0,303 | 0.4998 |
| MRT | **40 - 60** | heterophoria | 8,250±1,333; 4,625±0,865 | 0.095 |
| MRT | **40 - 100** | heterophoria | 8,250±1,333; 3,250±0,773 | 0.0034 |
| MRT | **60 - 100** | heterophoria | 4,625±0,865; 3,250±0,773 | 0.4052 |
| CT | 40 | **control - heterophoria** | 2,125±0,671; 9,000±1,414 | 0.0006 |
| CT | 60 | **control - heterophoria** | 0,750±0,342; 4,625±1,084 | 0.0032 |
| CT | 100 | **control - heterophoria** | 2,125±0,671; 2,625±0,980 | 0.0425 |
| MRT | 40 | **control - heterophoria** | 2,375±0,770; 8,250±1,333 | 0.0045 |
| MRT | 60 | **control - heterophoria** | 1,000±0,353; 4,625±0,865 | 0.0082 |
| MRT | 100 | **control - heterophoria** | 0,625±0,303; 3,250±0,773 | 0.0095 |

Table 5: Statistical information on the contrast threshold for each group in the Pilot study. In each row, the two parameters in bold represent the two conditions with which the statistical analysis is compared. The evaluation was done using post-hoc analysis of 3-way ANOVA. H stands for horizontal and V for vertical.

| Meridians | Viewing distance | Groups | Mean±SE | P-value |
| --- | --- | --- | --- | --- |
| **H - V** | 40 | control | 0,781±0,012; 0,791±0,016 | 0.8679 |
| **H - V** | 60 | control | 0,628±0,052; 0,614±0,054 | 0.8641 |
| **H - V** | 100 | control | 0,577±0,031; 0,548±0,037 | 0.8097 |
| **H - V** | 40 | heterophoria | 0,810±0,024; 0,791±0,032 | 0.8097 |
| **H - V** | 60 | heterophoria | 0,651±0,048; 0,672±0,050 | 0.8097 |
| **H - V** | 100 | heterophoria | 0,565±0,031;  0,590±0,047 | 0.8097 |
| H | **40 - 60** | control | 0,781±0,012; 0,628±0,052 | 0.0119 |
| H | **40 - 100** | control | 0,781±0,012; 0,577±0,031 | 0.0006 |
| H | **60 - 100** | control | 0,628±0,052; 0,577±0,031 | 0.8097 |
| H | **40 - 60** | heterophoria | 0,810±0,024; 0,651±0,048 | 0.0095 |
| H | **40 - 100** | heterophoria | 0,810±0,024; 0,565±0,031 | >0.0001 |
| H | **60 - 100** | heterophoria | 0,651±0,048; 0,565±0,031 | 0.3948 |
| V | **40 - 60** | control | 0,791±0,016; 0,614±0,054 | 0.0031 |
| V | **40 - 100** | control | 0,791±0,016; 0,548±0,037 | >0.0001 |
| V | **60 - 100** | control | 0,614±0,054; 0,548±0,037 | 0.6435 |
| V | **40 - 60** | heterophoria | 0,791±0,032; 0,672±0,050 | 0.0875 |
| V | **40 - 100** | heterophoria | 0,791±0,032; 0,590±0,047 | 0.0007 |
| V | **60 - 100** | heterophoria | 0,672±0,050; 0,590±0,047 | 0.4307 |
| H | 40 | **control - heterophoria** | 0,781±0,012; 0,810±0,024 | 0.8097 |
| H | 60 | **control - heterophoria** | 0,628±0,052; 0,651±0,048 | 0.8097 |
| H | 100 | **control - heterophoria** | 0,577±0,031; 0,565±0,031 | 0.8679 |
| V | 40 | **control - heterophoria** | 0,791±0,016; 0,791±0,032 | 0.9939 |
| V | 60 | **control - heterophoria** | 0,614±0,054; 0,672±0,050 | 0.6367 |
| V | 100 | **control - heterophoria** | 0,548±0,037; 0,590±0,047 | 0.8097 |

Table 6 : Clinical orthoptic information on the enrolled subjects: F female, M male, ortho orthophoria, X exophoria, E esophoria, RH Right hyperphoria, LH Left hyperphoria, OD right eye, and OS left eye.

| **Subject** | **Gender** | **Age** | **Group** | **Cover test** | | | | **Maddox rod test** | | | | **Dominant eye** | **Stereoscopic vision (arcsec)** | **Convergence** | | **NPC** |
| --- | --- | --- | --- | --- | --- | --- | --- | --- | --- | --- | --- | --- | --- | --- | --- | --- |
|  |  |  |  | **4m** | **1m** | **60cm** | **40 cm** | **4m** | **1m** | **60cm** | **40 cm** |  |  | **4m** | **40cm** |  |
| S1 | F | 34 | Heterophoria | E10 | E8 | E6 | E'6 | E10 | E8 | E6 | E'6 | OD | 40 | 40 | 40 | 3 |
| S2 | F | 34 | Heterophoria | E8 | E6 | - | E'6 | E6 | E6 | - | E'4 | OD | 40 | 25 | 30 | 4 |
| S3 | F | 29 | Heterophoria | X4 | X6 | - | X'12 | X4 | X10 | - | X'10 | OS | 40 | 18 | 20 | 6 |
| S4 | F | 22 | Heterophoria | X6 | X8 | - | X'4 | X6 | X6 | - | X'2 | OD | 40 | 16 | 25 | 7 |
| S5 | F | 20 | Heterophoria | X4 | X4 | - | X'10 | X4 | X6 | - | X'12 | OD | 40 | 14 | 10 | 10 |
| S6 | F | 29 | Heterophoria | E2 | E6 RH2 | - | E'8 RH'2 | E2 | E12 RH2 | - | E'25 RH' 2 | OD | 40 | 25 | 35 | 4 |
| S7 | F | 27 | Heterophoria | ortho | ortho | E2 | E'6 | ortho | E1 | E2 | E'6 | OD | 40 | 18 | 40 | 4 |
| S8 | F | 19 | Heterophoria | X4 | X4 | X10 | X'16 | X4 | X4 | X8 | X'12 | OD | 40 | 14 | 8 | 9 |
| S9 | M | 33 | Heterophoria | X2 | X3 | X7 | X'10 | X2 | X3 | X7 | X'10 | OD | 40 | 18 | 10 | 12 |
| S10 | M | 33 | Heterophoria | X2 | X4 | X6 | X'8 | X2 | X4 | X6 | X'8 | OS | 40 | 20 | 18 | 10 |
| S11 | M | 24 | Heterophoria | X2 | X2 | X2 | X'14 RH'2 | X2 | X2 | X2 | X'14 RH'2 | OD | 40 | 20 | 18 | 10 |
| S12 | M | 34 | Heterophoria | ortho | ortho | X2 | X'6 | X2 | X2 | X4 | X'8 | OD | 40 | 8 | 8 | 13 |
| S13 | F | 32 | Heterophoria | ortho | ortho | X2 | X'4 | E2 | E2 | E2 | E'2 | OD | 40 | 20 | 20 | 10 |
| S14 | F | 26 | Control | ortho | ortho | - | X'2 | ortho | X2 | - | X'4 | OD | 40 | 14 | 30 | 5 |
| S15 | F | 22 | Control | X4 | X4 | - | X'4 | X4 | X4 | - | X'4 | OS | 40 | 18 | 35 | 5 |
| S16 | F | 25 | Control | X2 | X2 | - | X'4 | X1 | X2 | - | X'4 | OD | 40 | 18 | 25 | 6 |
| S17 | F | 27 | Control | ortho | X2 | - | X'4 | ortho | X1 | - | X'5 | OD | 40 | 20 | 25 | 5 |
| S18 | M | 24 | Control | ortho | ortho | - | ortho | E1 | E1 | - | X'4 | OS | 40 | 16 | 30 | 9 |
| S19 | F | 28 | Control | ortho | X2 | X2 | X'4 | ortho | X2 | X2 | X'4 | OD | 40 | 20 | 40 | 5 |
| S20 | M | 26 | Control | ortho | ortho | ortho | ortho | E1 | E1 | ortho | ortho | OD | 40 | 25 | 35 | 4 |
| S21 | F | 33 | Control | ortho | ortho | ortho | X'2 | ortho | ortho | LH1 | LH'1 | OD | 40 | 14 | 25 | 5 |
| S22 | F | 23 | Control | ortho | ortho | X2 | X'4 | ortho | ortho | X2 | X'5 | OD | 40 | 18 | 30 | 4 |
| S23 | F | 20 | Control | X2 | X2 | X2 | X'4 | X2 | X2 | X2 | X'5 | OD | 40 | 18 | 30 | 5 |
| S24 | F | 35 | Control | ortho | ortho | ortho | X'2 | ortho | RH1 | RH1 | RH'1 | OD | 40 | 14 | 25 | 5 |
| S25 | M | 33 | Control | ortho | ortho | ortho | ortho | ortho | X2 | X2 | X'4 | OS | 40 | 14 | 20 | 7 |
| S26 | M | 23 | Control | ortho | ortho | ortho | ortho | ortho | ortho | ortho | X'1 | OS | 40 | 12 | 20 | 10 |

Table 7: Details of the experimental design. CD contrast detection, LM lateral masking, ET eye tracker, SF spatial frequency, AFC alternative forced choice, and CPD cycles per degree.

| **Experiment** | **Task** | **Target flanker separations (**λ) | **Viewing distance (m)** | **Viewing**  **Mode** | **Orientation** | **Nmax trials** | **N repetitions** | **SF (cpd)** | **Method** | **Presentation Time** |
| --- | --- | --- | --- | --- | --- | --- | --- | --- | --- | --- |
| Pilot study | CD + LM | 3,4, 6 | 0.4, 1 | Binocular | 0°, 90° | 80 | 2 | 4 | 2AFC | 80ms |
| 1 | CD + LM | 2,3,4,6 | 0.4, 0.6, 1 | Binocular | 0°, 90° | 80 | 2 | 4 | 2AFC | 80ms |
| 2 | CD + LM | 2,3,4,6 | 1 | Binocular and Monocular | 0°, 90° | 80 | 2 | 4 | 2AFC | 80ms |
| 3 | CD+LM+ET | 3 | 1 | Binocular | 0° | 80 | 3 | 4 | 2AFC | 80ms |

## **References:**

1. Howard, I. Binocular correspondence and the Horopter. in *Seeing in Depth, Vol I* (ed. Porteous, I.) 1–40 (2002).

2. Hillis, J. M. & Banks, M. S. Are corresponding points fixed? *Vision Res.* **41**, 2457–2473 (2001).

3. Von Noorden, G. K. & Campos, E. C. *Binocular vision and ocular motility : theory and management of strabismus*. (Mosby, 2002).

4. Kumar Morya, A., Solanki, K., Bhandari, S. & Naidu, A. Binocular Functions. in *Eye Motility* (IntechOpen, 2019). doi:10.5772/intechopen.84162

5. Scheiman, M. & Wick, B. *Clinical management of binocular vision : heterophoric, accommodative, and eye movement disorders*. (Wolters Kluwer/Lippincott Williams & Wilkins, 2014).

6. Aslin, R. N. & Dumais, S. T. Binocular vision in infants: a review and a theoretical framework. *Adv. Child Dev. Behav.* **15**, 53–94 (1980).

7. Coakes, R. L., Clothier, C. & Wilson, A. Binocular reflexes in the first 6 months of life: preliminary results of a study of normal infants. *Child. Care. Health Dev.* **5**, 405–408 (1979).

8. Birch, E. E., Gwiazda, J. & Held, R. The development of vergence does not account for the onset of stereopsis. *Perception* **12**, 331–336 (1983).

9. Worth, C. *Worth and Chavasse’s Squint : the binocular reflexes and the treatment of strabismus.* (Baillière Tindall & Cox, 1959).

10. Cacho-Martínez, P., García-Muñoz, Á. & Ruiz-Cantero, M. T. Do we really know the prevalence of accomodative and nonstrabismic binocular dysfunctions? *J. Optom.* **3**, 185 (2010).

11. Hashemi, H. *et al.* Binocular vision disorders in a geriatric population. *Clin. Exp. Optom.* (2021). doi:10.1080/08164622.2021.1922065

12. Hashemi, H. *et al.* The Prevalence of Strabismus, Heterophorias, and Their Associated Factors in Underserved Rural Areas of Iran. *Strabismus* **25**, 60–66 (2017).

13. Sánchez-González, M. C. et al. The effect of age on binocular vision normative values. J. Pediatr. Ophthalmol. Strabismus **57**, 363–371 (2020).

14. Walline, J. J., Mutti, D. O., Zadnik, K. & Jones, L. A. Development of phoria in children. *Optom. Vis. Sci.* **75**, 605–10 (1998).

15. Leone, J. F. *et al.* Prevalence of heterophoria and associations with refractive error, heterotropia and ethnicity in Australian school children. *Br. J. Ophthalmol.* **94**, 542–546 (2010).

16. Spierer, A. & Hefetz, L. Normal heterophoric changes: 20 years’ follow-up. *Graefe’s Arch. Clin. Exp. Ophthalmol.* **235**, 345–348 (1997).

17. Goss, D. A. & Jackson, T. W. Clinical Findings before the Onset of Myopia in Youth: 3. Heterophoria. *Optom. Vis. Sci.* **73**, 269–278 (1996).

18. Leat, S. J. *et al.* Binocular vision and eye movement disorders in older adults. *Invest. Ophthalmol. Vis. Sci.* **54**, 3798–3805 (2013).

19. Palomo Álvarez, C., Puell, M. C., Sánchez–Ramos, C. & Villena, C. Normal values of distance heterophoria and fusional vergence ranges and effects of age. *Graefe’s Arch. Clin. Exp. Ophthalmol.* **244**, 821–824 (2006).

20. Adler, F. H. & Hart, W. M. *Adler’s physiology of the eye : clinical application*. (Mosby Year Book, 1992).

21. Guide for Aviation Medical Examiners. Available at: https://www.faa.gov/about/office_org/headquarters_offices/avs/offices/aam/ame/guide/app_process/exam_tech/item54/amd/.

22. Dolman, P. The Maddox Rod Screen Test. *Trans. Am. Ophthalmol. Soc.* **17**, 235–49 (1919).

23. Rainey, B. B., Schroeder, T. L., Goss, D. A. & Grosvenor, T. P. Inter-examiner repeatability of heterophoria tests. *Optom. Vis. Sci.* **75**, 719–26 (1998).

24. Polat, U. & Sagi, D. Lateral interactions between spatial channels: Suppression and facilitation revealed by lateral masking experiments. *Vision Res.* **33**, 993–999 (1993).

25. Polat, U. & Sagi, D. *The Architecture of Perceptual Spatial Interactions Lateral masking*. *Vision Res* **34**, 73-78 (1994).
